# Visualizing Molecular-Scale 3D Distributions of Ionic Liquids in Electric Double-Layer Capacitor by 3D Scanning Force Microscopy with Variable Tip/Sample Bias Voltages

**DOI:** 10.1021/acsami.5c11718

**Published:** 2025-09-06

**Authors:** Takahiko Ikarashi, Takashi Sumikama, Kaito Hirata, Ryo Sakakibara, Takumi Yoshino, Kazuki Miyata, Keisuke Miyazawa, Sunao Shimizu, Yoshihiro Iwasa, Takeshi Fukuma

**Affiliations:** † Division of Nano Life Science, 12858 Kanazawa University, Kakuma-machi, 920-1192 Kanazawa, Japan; ‡ WPI Nano Life Science Institute (WPI-NanoLSI), Kanazawa University, Kakuma-machi, 920-1192 Kanazawa, Japan; § Department of Electronics, Graduate School of Engineering, Nagoya University, Furo-cho, Chikusa-ku, Nagoya 464-8603, Japan; ∥ Division of Electrical Engineering and Computer Science, Kanazawa University, Kakuma-machi, 920-1192 Kanazawa, Japan; ⊥ Faculty of Frontier Engineering, Kanazawa University, Kakuma-machi, 920-1192 Kanazawa, Japan; # Faculty of Engineering, 57948 Toyama Prefectural University 939-0398, Toyama, Japan; ¶ Quantum-Phase Electronics Center (QPEC) and Department of Applied Physics, The University of Tokyo, Tokyo 113-8656, Japan; ∇ 13593 RIKEN Center for Emergent Matter Science (CEMS), 2-1, Hirosawa, Wako 351-0198, Saitama, Japan; 9 Department of Physical Science and Engineering, Nagoya Institute of Technology, Nagoya 466-8555, Japan

**Keywords:** atomic force microscopy, three-dimensional scanning force microscopy, ionic liquid, electric double layer, electric double layer capacitor

## Abstract

Atomic force microscopy (AFM) imaging of ionic liquid (IL) distribution in electric double-layer (EDL) devices has been actively explored to understand the origin of their excellent performance. However, this has been impeded by insufficient resolution or a poor understanding of the mechanisms of 3D IL imaging. Here, we overcome these difficulties using 3D scanning force microscopy (3D-SFM) with variable tip/sample bias voltages for visualizing 3D *N*,*N*-diethyl-*N*-methyl-*N*-(2-methoxyethyl)­ammonium bis­(trifluoromethanesulfonyl)­imide (DEME-TFSI) distributions on a Au electrode in EDL capacitors. Unlike previous reports, the multilayered vertical IL distribution and lateral molecular arrangements in the first adsorption layer are simultaneously visualized in one 3D image. This has allowed us to find the sample-bias-dependent changes in the molecular stability and thickness of the first IL adsorption layer, suggesting the significant bias dependence of the EDL capacitance. Such bias dependence is also confirmed by our molecular dynamics simulation and electrochemical impedance spectroscopy experiments, demonstrating the capability of 3D-SFM to provide molecular insights into the macroscopic device properties. Detailed comparisons between simulation and experiments also reveal that the 3D-SFM force contrasts mostly represent the distribution of anions having a higher molecular weight, yet the contrast is strongly enhanced by a positive tip bias. This is because the positively (or negatively) charged Au-coated tip is covered with a quasi-solid-state anion (or cation) layer, enhancing (or reducing) the electrostatic repulsion from the anions in the EDL. This counterintuitive finding should reinforce the theoretical basis for 3D IL imaging and help understand the molecular-scale origins of the EDL device performance.

## Graphical abstract

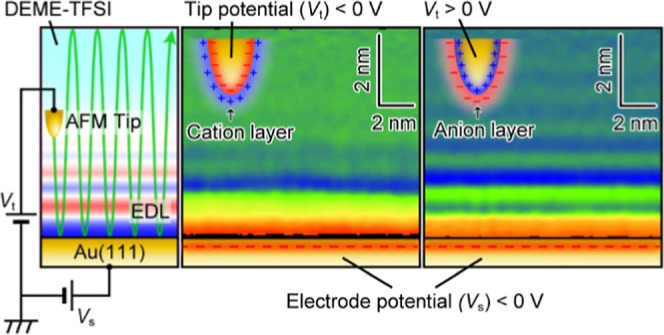

## Introduction

An electric double layer (EDL) formed at an electrode–electrolyte interface significantly influences various phenomena such as corrosion,^1^ molecular adhesions,^2^ and devices such as EDL capacitors,^3,4^ transistors,^5^ and superconductors.^6^ Especially, EDL transistors (EDLTs) using ionic liquids (ILs) instead of solid insulators in field-effect transistors (FETs) have attracted much attention. Due to the formation of an EDL with a molecular-scale thickness at the IL/solid interface, the EDLTs exhibit charge densities several tens of times higher than those of conventional FETs at low gate voltages (<1 V).^7–9^ Such a high charge density has enabled voltage control of various interfacial properties and phenomena such as ferromagnetism,^10^ superconductivity,^8,11–13^ and metal–insulator transition.^14^ However, the correlation between these properties and the interfacial IL structures in the EDL has not been well understood at the molecular level. This has hindered the improvement of device performance, practical applications and the development of related fields.

Various experimental techniques have been used to investigate the interfacial IL structures. For instance, interface analysis techniques with a high vertical resolution, such as X-ray^15–17^ and neutron reflectometry,^18^ sum frequency generation vibration spectroscopy,^19–21^ surface-enhanced Raman spectroscopy^22–24^ and surface force apparatus^25,26^ have revealed the formation of periodic multilayer structures at the interface. However, these methods provide averaged information over a micrometer-scale area; hence, any lateral inhomogeneity may be overlooked. In contrast, atomic force microscopy (AFM)^27–38^ uses a tip with a radius of a few nanometers to locally measure layered IL distributions at the interface.

Recently, in-liquid three-dimensional AFM (3D-AFM) has been developed by several groups^39^ and used for imaging EDL structures at the solid–liquid interfaces.^40–44^ This technology has also been applied to IL/solid interface measurements.^45^ While these studies focused on the vertical distribution of the multilayer structures, the in-plane ion distribution has not been discussed in detail. Meanwhile, two-dimensional AFM (2D-AFM) (i.e., standard AFM) has been used for imaging the in-plane subnanoscale distribution of relatively immobile ions adsorbed on a solid surface.^46–49^ However, 2D-AFM cannot provide information on the vertical ion distribution. Although we can complementarily use one-dimensional (1D), 2D- and 3D-AFMs to probe the 3D organization of an interface,^50^ it is often difficult to correlate the independently obtained images with molecular-scale precision. Therefore, a true subnanoscale 3D-AFM measurement at the IL/solid interface is desired.

Among various 3D-AFM techniques, 3D scanning force microscopy (3D-SFM)^51^ is one of the most promising candidates to solve these problems. In this method, the vertical tip position is sinusoidally modulated during the lateral tip scan, and the force applied to the tip is recorded to produce a 3D force map. Combined with the frequency-modulation (FM) force-detection method (i.e., FM-AFM), a true subnanoscale 3D imaging capability of 3D-SFM has been demonstrated for various solid–water interfaces.^51–59^ While such 3D imaging has not yet been realized for a solid-IL interface or an electrochemical interface, FM-AFM was successfully used for atomic- or molecular-resolution 2D imaging in ILs^60–62^ and 1D measurements of the layered IL structures on several surfaces.^30,63–66^ These previous works suggest that 3D-SFM is very promising for a true 3D subnanoscale imaging of an IL structure.

The electrode potential dependence of the IL/electrode interface structure has been widely investigated by AFM.^27–29,31,45,67–70^ In contrast, the influence of the tip charges has hardly been investigated, although a large impact is expected. For the measurements of water–solid interfaces, previous experimental and theoretical studies revealed that the subnanoscale contrasts in the 3D force map largely represent the mass density distribution of water.^71^ Meanwhile, the contribution of tip charges should be considered in a dense ionic solution (∼5.5 M). For example, a previous 3D-AFM study on a dense electrolyte–mica interface reported that a 3D force map obtained with a neutral tip represents a mass density distribution while a charged tip provides a charge density distribution.^72^ In addition, a theoretical study, where forces measured with charged and uncharged tips in IL were calculated, reported that a force curve measured with a positively charged tip reflects the cation distribution while a negatively charged tip provides an anion distribution.^73^ However, in previous IL studies by 3D-AFM, the tip charge or potential has not been controlled, so that its effect has not been well understood.

In this study, we have measured the *N*,*N*-diethyl-*N*-methyl-*N*-(2-methoxyethyl)­ammonium bis­(trifluoromethanesulfonyl)­imide (DEME-TFSI)/Au(111) electrode interface structures with subnanoscale resolution by 3D-SFM with variable tip and sample bias voltages. Furthermore, we have performed molecular dynamics (MD) simulations to calculate the sample bias dependence of the DEME-TFSI/Au(111) interface structures. By comparing the simulated results with the experiments, we clarify the effects of the tip and sample bias voltages on the interface structure and the molecular-scale contrasts observed by 3D-SFM.

## Results and Discussions

### 3D-SFM Imaging and MD Simulation

The IL used in this study was DEME-TFSI (Figure 1a). This IL has been widely investigated as a material of electrochemical devices such as EDL capacitors and transistors (EDLC and EDLT) due to its wide potential window (Figure 1b), excellent stability against temperature, and ability to provide high EDL capacitance.^74–80^ The molecular weight (MW) of TFSI^–^ (280.147) is nearly twice as high as that of DEME^+^ (146.253). An EDLC consisting of two Au(111) electrodes was prepared as a sample (Figure 1c). The cantilever was coated with Au by the sputtering method to ensure electrical conductivity. During the 3D-SFM measurement (Figure 1d), one of the two electrodes constituting the capacitor was grounded, and the potential of the other electrode (*V*_s_) and tip (*V*_t_) was controlled with respect to the ground electrode.

**Figure 1:**
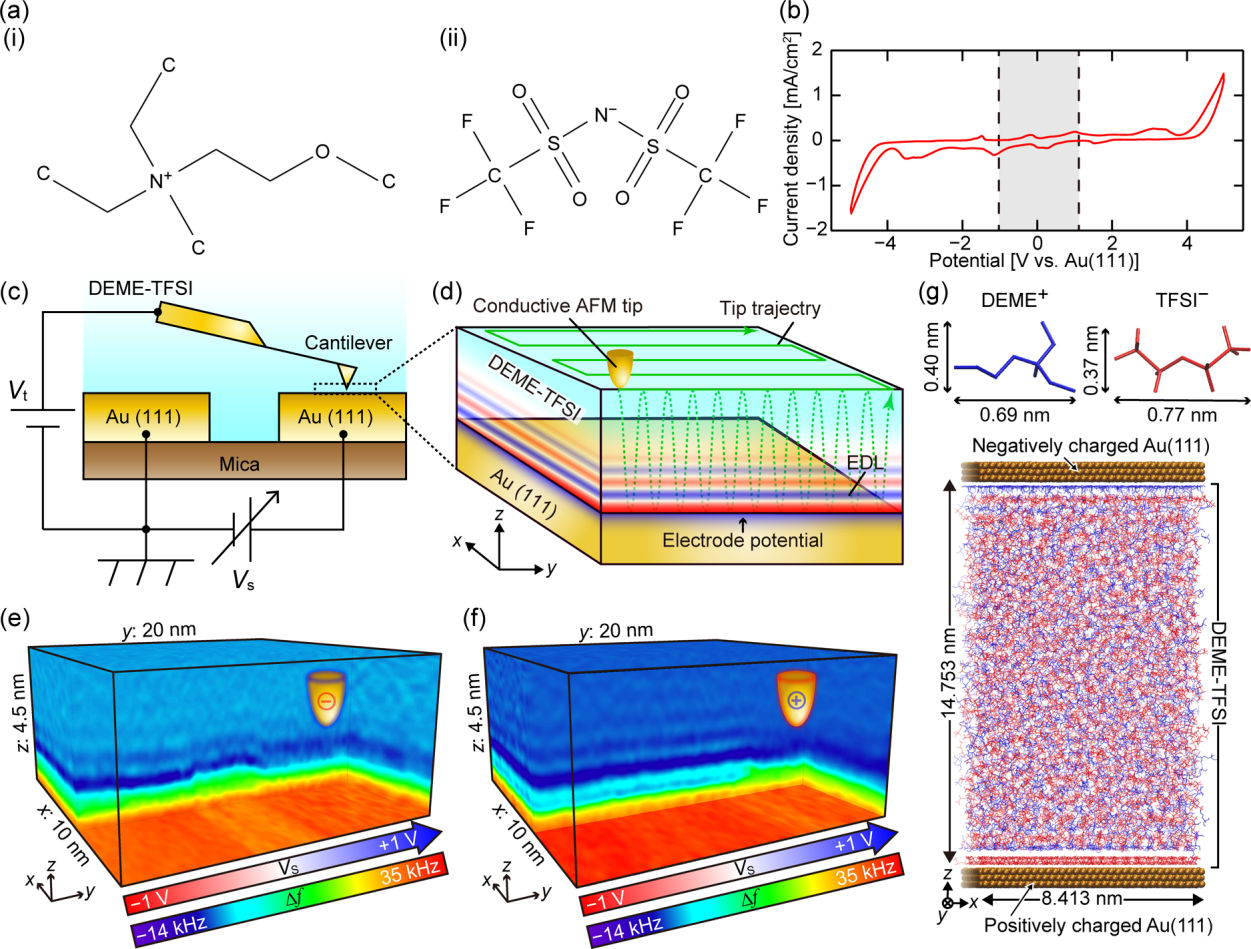
Chemical structure and potential window of the IL and methods for the 3D-SFM measurements and MD simulations. (a) Chemical structures of (i) DEME^+^ and (ii) TFSI^–^. (b) CV curve measured in DEME-TFSI on the Au(111) electrode. (c,d) Schematics showing the 3D-SFM setup and measurement principle, respectively. (e, f) 3D-SFM images obtained with *V*_t_ of −0.5 V and +0.5 V, respectively. The *V*_s_ was swept from −1 V to +1 V during the 3D-SFM measurements. In the 3D-SFM images, *V*_s_ changes along the *y* axis (i.e., slow scan direction). (g) Snapshot of the MD simulation model. Blue and red molecules are DEME^+^ and TFSI^–^, respectively. Charges with a density of +24 μC/cm^2^ and −24 μC/cm^2^ were given to the top and bottom electrodes, respectively.

Figure 1b shows a cyclic voltammetry (CV) curve measured in DEME-TFSI with the Au(111) electrode using the setup shown in Figure 1c. While the CV curve shows an almost flat profile at the voltage range from −4 V to +4 V, a large current was observed outside this range, suggesting that this range corresponds to the potential window of DEME-TFSI. Although some peaks are observed in this range, previous studies reported that some peaks originate from the adsorption and desorption of ions at the interface.^27,81,82^ We performed 3D-SFM measurements within the potential range from −1 V to +1 V as indicated by the gray background in Figure 1b to ensure that the experiments were performed within the potential window. Meanwhile, *V*_t_ was set at −0.5 V or +0.5 V during the 3D-SFM measurements.

In previous AFM studies, 1D force curves were measured at a fixed position to investigate the electrode potential dependence of the IL structures.^27,28,31^ However, the force curve profile is sensitive to the tip/sample drift and tip changes. Thus, it is difficult to ensure that the observed force changes are caused only by the potential changes. Although the 3D-SFM is not completely free from these problems, it is possible to discriminate these changes from the site-dependent changes, providing a higher reliability.

To measure the *V*_s_ dependence of the IL structure at the DEME-TFSI/Au(111) interface, *V*_s_ was swept from −1 V to +1 V during the 3D-SFM measurement (Figure 1e,f). The *V*_s_ sweep was synchronized with the 3D-SFM scan in the *y* direction. Therefore, *V*_s_ varies along the *y* axis. *V*_t_ was controlled at −0.5 V for Figure 1e and at +0.5 V for Figure 1f. These images reveal the difference in the *V*_s_ dependence of the molecular-scale IL structure observed with different *V*_t_.

MD simulations were performed to understand the molecular-scale mechanisms of the *V*_s_ dependence. A simulation model consists of two opposing parallel Au(111) electrodes and DEME^+^ and TFSI^–^ ions with a density equal to the bulk value (1.41 g/cm^3^)^109^ placed between them (Figure 1g). A force field for DEME^+^ and TFSI^–^ was developed by scaling charge and sigma of the Lennard-Jones interaction to simultaneously match the density and diffusion coefficient with experiments (see Methods and Figure S12). In the experiments (Figure 1c), *V*_s_ applied between the Au(111) electrodes was controlled. However, since it is difficult to directly define the electrode potential in the simulation, we placed the same amount of charges with opposite polarities at the two opposing electrode surfaces. The surface charge density σ_s_ was varied from −28 to +28 μC/cm^2^. The simulation was run for 500 ns after initial equilibration, and the collected data was averaged to obtain 3D density distributions of ions and charges. From the charge density distribution, the voltage drops at the electrode interfaces were estimated by Poisson’s equation, and the relationship between σ_s_ and the sample bias voltage was determined as σ_s_ = ∼ 26.72 μC/cm^2^ for a sample bias of 1 V (see Figure S1). Meanwhile, we also experimentally estimated the corresponding value as ∼ 24.65 μC/cm^2^ (Figure S2), which approximately agrees with the value obtained by the simulation. Therefore, here we define the bias voltage *V*_s_^*^ indued by σ_s_ in the simulation as follows.

$$V_{s}^{*}=\sigma_{s}/26.72\,\;\left[V\right]$$ 1

### Vertical Ion Distributions

[Figure 2a,b­(i)] show the *yz* cross sections of Figure 1e,f averaged over the *x* direction, respectively. For quantitative discussions, the frequency shift (Δ*f*) was converted to the force using the Sader equation.^83^ First, we discuss the features commonly observed at the positive and negative *V*_t_. [Figure 2a,b­(i)] show that the interfacial structure dramatically changes at a certain *V*_s_. In this measurement, a reference electrode was not used because the sample configuration was designed to mimic an EDLC device consisting of two electrodes. Therefore, it is difficult to directly compare *V*_s_ values between different experiments or between experiments and simulations. Instead, we defined Δ*V*_s_ as a potential relative to the *V*_s_ value of the dramatic change in the IL structure because it was commonly observed in all the experiments.

**Figure 2:**
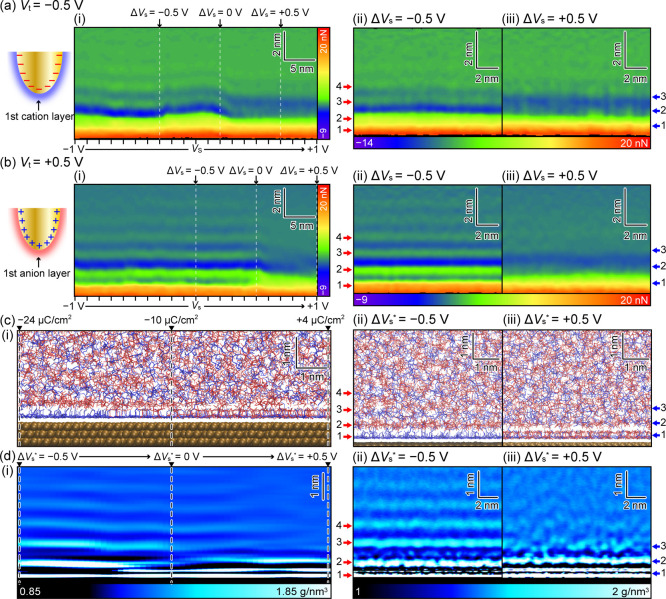
3D-SFM measurements and MD simulation of the DEME-TFSI/Au(111) electrode interface structure. (a,b) (i) *yz* cross-section averaged over *x* direction in Figure 1e. (ii,iii) *xz* cross sections at (ii) Δ*V*_s_ = −0.5 V and (iii) Δ*V*_s_ = +0.5 V. (a) *V*_t_ = −0.5 V. (b) *V*_t_ = +0.5 V. (c) (i) σ_s_ dependence of the snapshot of the MD simulation model. (ii,iii) Snapshots of MD simulations at (ii) Δ*V*_s_* = −0.5 V (σ_s_ = −24 μC/cm^2^) and (iii) + 0.5 V (+4 μC/cm^2^). Blue and red molecules represent DEME^+^ and TFSI^–^, respectively. (d) (i) σ_s_ dependence of the ion density *z* profiles calculated at each σ_s_ value by *xy*-averaging the 3D ion density map. (ii,iii) 2D projections of the 3D ion density maps calculated with (ii) Δ*V*_s_* = −0.5 V (σ_s_ = – 24 μC/cm^2^) and (iii) + 0.5 V (+4 μC/cm^2^). The red and blue arrows indicate the expected *z* positions of the individual IL layers. See Figure S3 for details on how to prepare c (i) and d (i).

To clarify the Δ*V*_s_ dependent differences, *xz* cross sections at Δ*V*_s_ = −0.5 V and +0.5 V were extracted from the 3D images [Figure 2a,b­(ii,iii)]. [Figure 2c,d­(i)] show the σ_s_ dependence of the snapshots of the MD simulation model and the ion density *z* profiles calculated at each σ_s_ value by *xy*-averaging the 3D ion density map, respectively. The simulation was performed for σ_s_ = −28 ∼ + 28 μC/cm^2^, and all the results are shown in Figure S3a. The simulation results suggest that a layered structure can be formed even with a positive bias if a voltage of +1 V or higher is applied. In this experiment, problems such as the occurrence of electrochemical reactions and the movement of gold atoms on the gold substrate surface causing roughening and reconstitution were often observed when voltages exceeding +1 V were applied. To minimize the risk of such phenomena, which are not fully accounted for by the MD simulation, we conducted the experiment in this bias range (Figure 1b), which is reproducible and safe for the experiment.

Among them, the results for σ_s_ = −24 ∼ + 4 μC/cm^2^ are shown in [Figure 2c­(i)], where the dramatic change in the IL structure is observed near the center of the σ_s_ axis (−10.07 μC/cm^2^). Similar to Δ*V*_s_ for the experiments, we define a potential with respect to this σ_s_ value (−10.07 μC/cm^2^) as Δ*V*_s_*. As discussed above, 26.72 μC/cm^2^ surface charges correspond to the 1 V bias application. Thus, the relationship between Δ*V*_s_* and σ_s_ is given by the following equation.

2 $$\Delta V_{s}^{*}=\left(\sigma_{s}+10.07\right)/26.72\left[V\right]$$

From this equation, we find that σ_s_ values of −24 μC/cm^2^ and +4 μC/cm^2^ indicated in Figure 2c,d approximately correspond to Δ*V*_s_* = −0.5 V and +0.5 V, respectively.

In the 3D-SFM experiments, at Δ*V*_s_ = −0.5 V, an IL structure with more than four layers was observed [Figure 2a,b­(ii)]. Meanwhile, at Δ*V*_s_ = +0.5 V, such a multilayered structure was not clearly observed [Figure 2a,b­(iii)]. In the MD simulation, at Δ*V*_s_* = −0.5 V, an IL structure with more than six layers was observed [Figure 2d­(ii)]. Meanwhile, two clear layers and one obscure layer on top of them were observed at Δ*V*_s_* = +0.5 V [Figure 2d­(iii)]. However, the large gap near the second layer position seen at Δ*V*_s_ = +0.5 V [Figure 2ab­(iii)] was not observed in the simulation [Figure 2d­(iii)]. The reason for this difference will be discussed later with the force curves shown in Figure 3. Except for this point, we found that the density distributions at Δ*V*_s_* = ± 0.5 V agreed well with the force maps at Δ*V*_s_ = ± 0.5 V, respectively.

**Figure 3:**
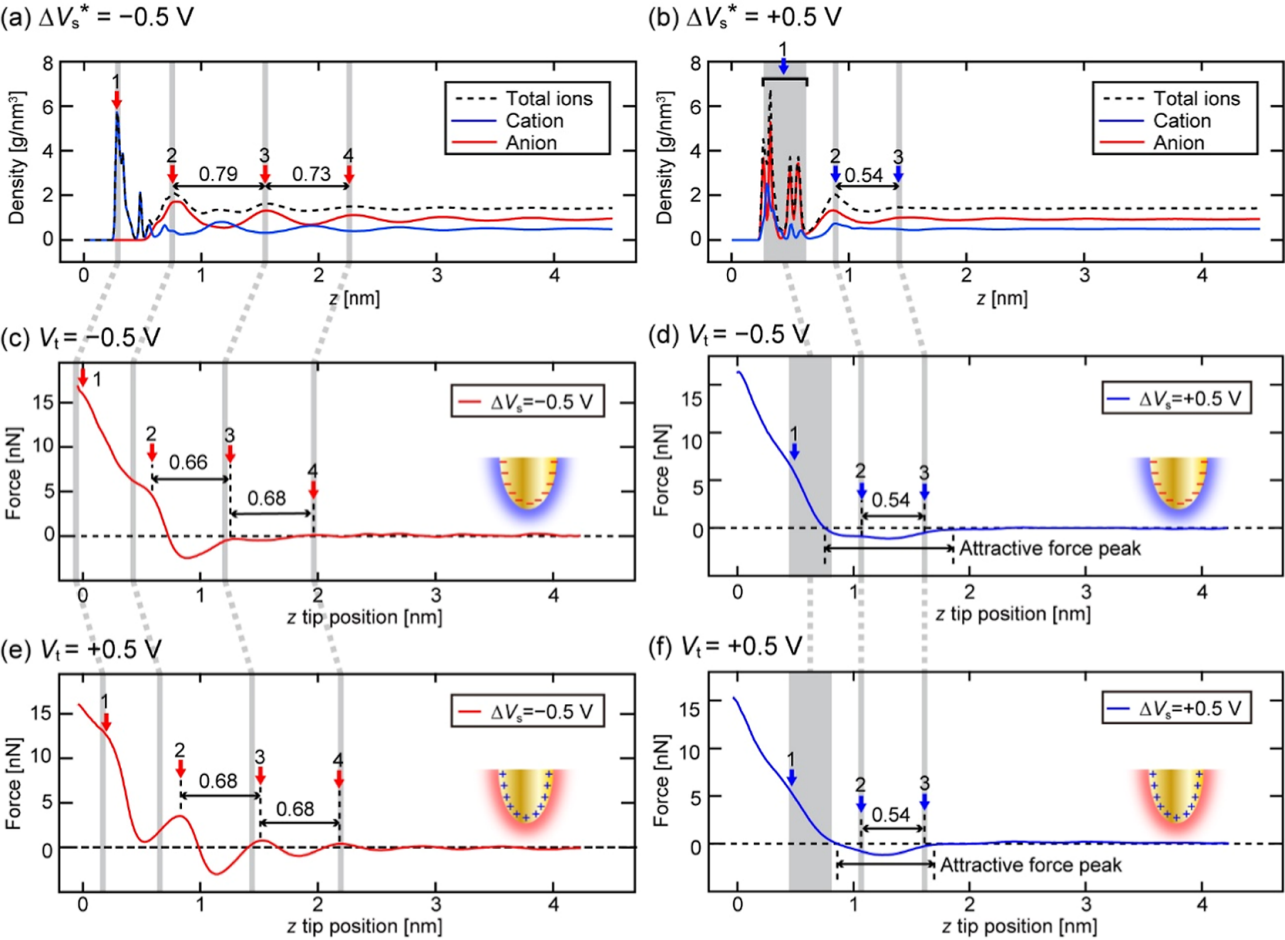
Comparison between the simulated ion density profiles and the experimentally measured force curves. (a,b) *z* density profiles of DEME^+^ (blue lines), TFSI^–^ (red lines), and total ions (black dotted lines) averaged over the *xy* plane at (a) Δ*V*_s_* = −0.5 V (σ_s_ = −24 μC/cm^2^) and (b) + 0.5 V (σ_s_ = +4 μC/cm^2^). (c-f) Force curves averaged over the *xz* cross sections shown in (c) Figure 2a­(ii), (d) Figure 2a­(iii), (e) Figure 2b­(ii), and (f) Figure 2b­(iii). The arrows indicate the position of the layers. The gray lines correspond to the relative peak positions of the total ion density profile obtained by the MD simulation. The red and blue arrows indicate the *z* position of the corresponding IL layer in Figure 2.

Figures 2a,b also reveal the difference in the force maps obtained with the positive and negative *V*_t_. For Δ*V*_s_ = −0.5 V [Figure 2a,b­(ii)], the layered structure is more clearly observed with *V*_t_ = +0.5 V than with *V*_t_ = −0.5 V. For example, the boundary between the first and second layers is clearly observed with *V*_t_ = +0.5 V but not with *V*_t_ = −0.5 V. In contrast, no significant dependence on *V*_t_ is observed for Δ*V*_s_ = +0.5 V [Figure 2a,b­(iii)], probably because no distinct layers were observed under this condition.

To understand the origin of the layered contrasts observed by 3D-SFM, here we analyze the *z* ion density profiles of the anions, cations, and total ions (i.e., a sum of them) obtained by the MD simulations and the experimentally obtained force curves. Figure 3a,b show the density profiles of DEME^+^ and TFSI^–^ and total ions averaged over the *xy* plane obtained at Δ*V*_s_* = ± 0.5 V. Figure 3c−f show the force curves averaged over the *xz* cross-section shown in [Figure 2a­(ii,iii), b­(ii,iii)], respectively.

In 3D-SFM measurements, both ion mass and charge density distributions should contribute to the force contrasts. Among them, first, we focus on the influence of mass density. In Figure 3a,b, the peak positions of the total ions largely correspond to those of the anions. This is probably because the anion’s MW (280.147) is approximately twice as large as that of the cations (146.253). The only exception is the first peak observed with a negative sample bias, where the first adsorption layer is dominated by the cations, so that the cation peak corresponds to the total ion peak.

The relative peak positions in the measured force curves largely agree with those in the simulated total ion density profile as indicated by the gray lines in Figure 3, except for the second peaks observed at Δ*V*_s_* = −0.5 V. Possible origins for this difference include the difference between the force and ion density profiles, the influence of the long-range background force, and tip deformation.^84,85^ However, the agreement of the majority of the peak positions suggests that the force contrasts largely represent the ion mass density distribution. This argument is consistent with the previous contact-mode AFM study,^86^ where experimentally measured force peak positions agreed well with the simulated density peak positions for the heavier ion.

At Δ*V*_s_* = +0.5 V, four narrow peaks are seen in the total ion density profile at the first layer position indicated by blue arrow 1 in Figure 3b. These peaks correspond to the O, S, N, and F atoms constituting TFSI^–^. This is because the first adsorption layer is so firmly fixed that the positions of the constituent atoms are separately visible even in the time-averaged density profile. At the second peak position, both anion and cation distributions show a positive peak, while only anion distribution shows a positive peak at the third peak position.

From the σ_s_ dependence of the MD simulation model shown in Figure S3a, we can see that alternating layers of the anions and cations are formed at σ_s_ = −26.21 ∼ −10.07 μC/cm^2^ (i.e., Δ*V*_s_* <0). In contrast, at σ_s_ = −10.07 ∼ + 26.21 μC/cm^2^ (i.e., Δ*V*_s_* >0), a gap is formed above the first adsorption layer, and the alternating layers are not clearly confirmed above it. Figure S3b shows the σ_s_ dependence of the total ion density *z* profile averaged over the simulation model. This density map also confirms a clearer layered structure at negative Δ*V*_s_* than at positive Δ*V*_s_*. This difference cannot be explained by the negative offset of σ_s_ observed at zero Δ*V*_s_*. This point will be later explained with the molecular orientation in the first adsorption layer shown in Figure 5.

The force curves obtained at Δ*V*_s_ = +0.5 V show relatively weak oscillations (Figure 3d,f), which is consistent with the above simulation results. In contrast to the results for the negative Δ*V*_s_, the distance between the second and third peaks in the measured force curves (∼0.54 nm for both *V*_t_ = ± 0.5 V) agrees well with that in the simulated density profile (Figure 3b). This better agreement may be explained by the smaller tip deformation due to the smaller oscillatory force or the weaker influence of the substrate due to the slightly higher peak positions.

The second peak of the force curves shown in Figure 3d,f appears in the long-range attractive force regime. This attractive regime is visualized as the large gap in the force map shown in [Figure 2a,b­(iii)]. As the second peak is located inside this gap, the weak contrast corresponding to the peak is almost invisible with the used color scale.

Generally, the distance between peaks in the force curve alone is insufficient to determine whether each layer is composed of cations or anions. It is also difficult to determine whether the peaks are assigned to the substrate or the first layer based only on the shape of the force curve. Here, we measured the continuous change in the IL structure induced by varying bias voltage in a single map, which allowed us to capture the relative position shift that occurs when the anion adsorbed layer transitions to the cation adsorbed one. Furthermore, according to MD simulation data, the first layer is extremely rigid and close to solid so that it is unlikely that the probe will penetrate this layer. In contrast, the second layer exhibits significantly weaker adsorption than the first layer. Therefore, it is reasonable to assume that the force applied in this experiment (exceeding 10 nN) could penetrate the second layer. Thus, in Figure 3, the penetrable layer is assigned as the second layer, and the nonpenetrable layer is assigned as the first layer. Therefore, it was concluded that only these peak assignments could explain the MD simulation results.

Next, we discuss the *V*_t_ dependence of the force curves. Figure 3c,e show that a clearer force oscillation is observed with the positive *V*_t_ than with the negative *V*_t_. As the force peaks correspond to the anion peaks, this result suggests that the positive *V*_t_ enhances the repulsive force exerted by the anions. This apparently counterintuitive result can be explained by considering the quasi-solid-state adsorption layer as follows.

In the MD simulation, molecules in the first adsorption layer hardly desorb from or diffuse on the Au substrate, suggesting their quasi-solid-state adsorption. Similarly, the Au-coated tip surface should be terminated with a rigid anion or cation layer with the positive or negative *V*_t_, respectively. With *V*_t_ = +0.5 V, the anion tip should enhance the repulsive force peaks received from the anion layer due to the electrostatic repulsive interaction. In contrast, the cation tip formed with *V*_t_ = −0.5 V should suppress the force peaks due to the attractive electrostatic interaction. Thus, a clearer layered structure was observed with the positive tip bias [Figure 2b­(ii)] than with the negative tip bias [Figure 2a­(ii)]. These results confirm that both ion mass density distribution and tip charges affect the force contrasts obtained by 3D-SFM.

Strictly speaking, the second and higher IL layers formed on the quasi-solid-phase first layer should also influence the force detected by the tip. However, they contribute in the same direction as the first layer. For example, at *V*_t_ > 0 V, cation­(2nd)/anion­(3rd)/cation­(4th) layers are formed on the quasi-solid-phase anion tip. If they overlap with anion­(3rd)/cation­(2nd)/anion­(1st) layers on the sample surface during the tip approach, the tip should feel a repulsive force. Similarly, overlapping with cation­(3rd)/anion­(2nd)/cation­(1st) layers should produce an attractive force. Since these force directions are the same as expected from the contribution of the quasi-solid-phase first adsorption layer on the tip, the contributions from the higher IL layers do not alter the above conclusion.

### In-Plain Ion Distribution

Next, we analyze the subnanoscale in-plane structures of the first adsorption layer. Figure 4 shows the iso-Δ*f* surfaces extracted from the 3D Δ*f* maps. The Δ*f* curves corresponding to the force curves in Figure 3c−f are shown in Figure S4a. The Δ*f* values giving the clearest iso-Δ*f* surface contrast were Δ*f* = +20 kHz for *V*_t_ = −0.5 V (Figure 4a) and Δ*f* = +19 kHz for *V*_t_ = +0.5 V (Figure 4b). The *z* feedback positions corresponding to these Δ*f* set points are around the first adsorption layer position, as indicated by the gray background in Figure S4a.

**Figure 4:**
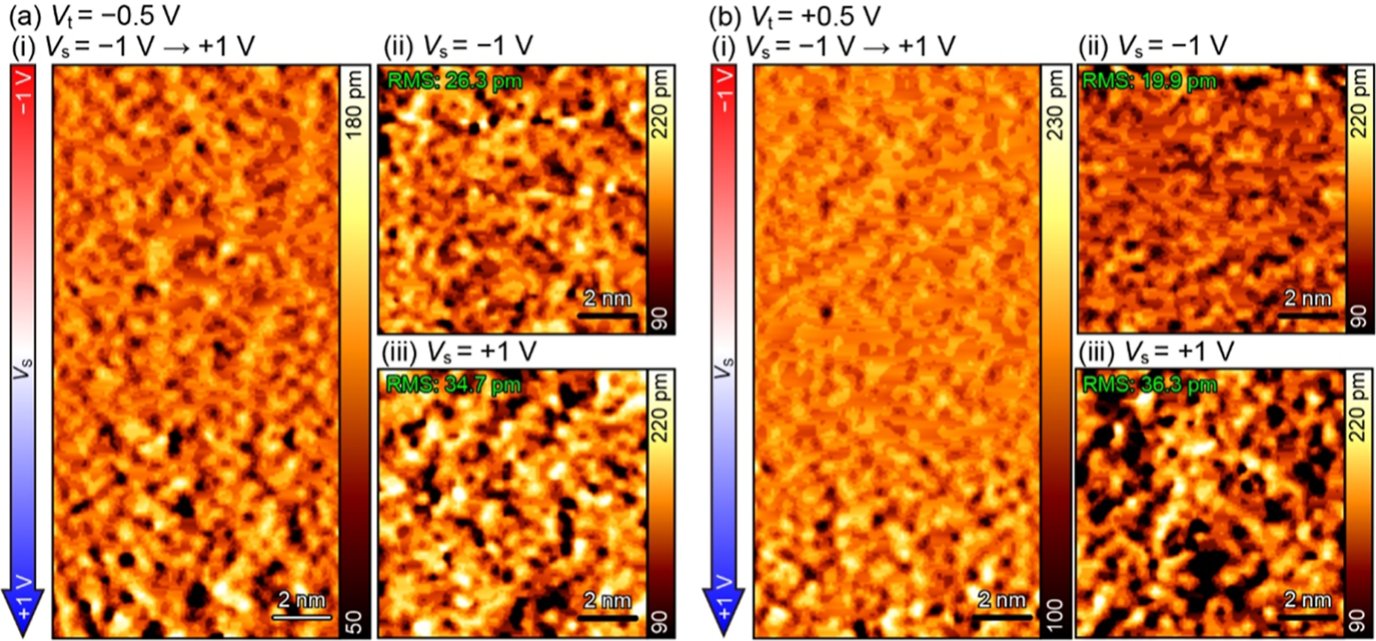
*V*_s_ dependence of the iso-Δ*f* surface extracted from the 3D-SFM images. (a) Δ*f* = +20 kHz, *V*_t_ = −0.5 V. (b) Δ*f* = +19 kHz, *V*_t_ = +0.5 V. (i) *V*_s_ was varied from −1 V to +1 V while the tip was scanned from the top to the bottom. (ii) *V*_s_ was kept constant at −1 V. (iii) *V*_s_ was kept constant at +1 V. The RMS roughness is shown in the upper left of (ii) and (iii).

All the iso-Δ*f* surfaces in Figure 4 show granular contrasts with a scale similar to the ion sizes of DEME^+^ and TFSI^–^ (i.e., 0.7–0.8 nm, Figure 1g). Thus, the observed subnanoscale contrasts should reflect the surface structure of the quasi-solid-state first adsorption layer. The molecular-scale contrast becomes clearer as *V*_s_ was swept from −1 V to +1 V [Figure 4a,b­(i)]. This dependence is also confirmed by comparing the images taken with a fixed *V*_s_ of ±1 V [Figure 4a,b­(ii,iii)]. Quantitatively, the RMS roughness increases from 26.3 to 34.7 pm for *V*_t_ = −0.5 V and from 19.9 to 36.3 pm for *V*_t_ = +0.5 V. In contrast to the *V*_s_ dependence, no significant *V*_t_ dependence was confirmed for these molecular-scale features.

It should be noted that the corrugation changes observed in the 2D *xy* images (Figure 4) are accompanied by the pronounced variations in the vertical IL distribution (Figure 2). Importantly, these structural changes are both reproducible and reversible, irrespective of the direction of the bias sweep, as demonstrated in Figure S5. Therefore, the observed features in Figure 4 are not attributable to tip-induced perturbations but rather represent bias-induced phenomena.

Although the vertical cross sections shown in Figure 2a,b were derived from the same 3D-SFM data used for producing Figure 4, the surface corrugations may not be clearly visible due to the averaging or relatively large *z* scale. However, these corrugations are clearly visible in the nonaveraged magnified images as demonstrated in Figure S6.

To understand the origin of the *V*_s_ dependence, the molecular orientation and stability in the first adsorption layer were analyzed with the MD simulation results. [Figure 5a­(i)] shows the snapshots of the first adsorption layer model simulated with various *V*_s_*. Note that the *V*_s_* values defined for the simulation should not be directly compared with the *V*_s_ values defined for the experiments. [Figure 5a­(ii)] shows the orientation distribution of DEME^+^ and TFSI^–^ in the first adsorption layer. The orientation angles (θ) of each ion are defined as shown in [Figure 5a­(iii)].

**Figure 5:**
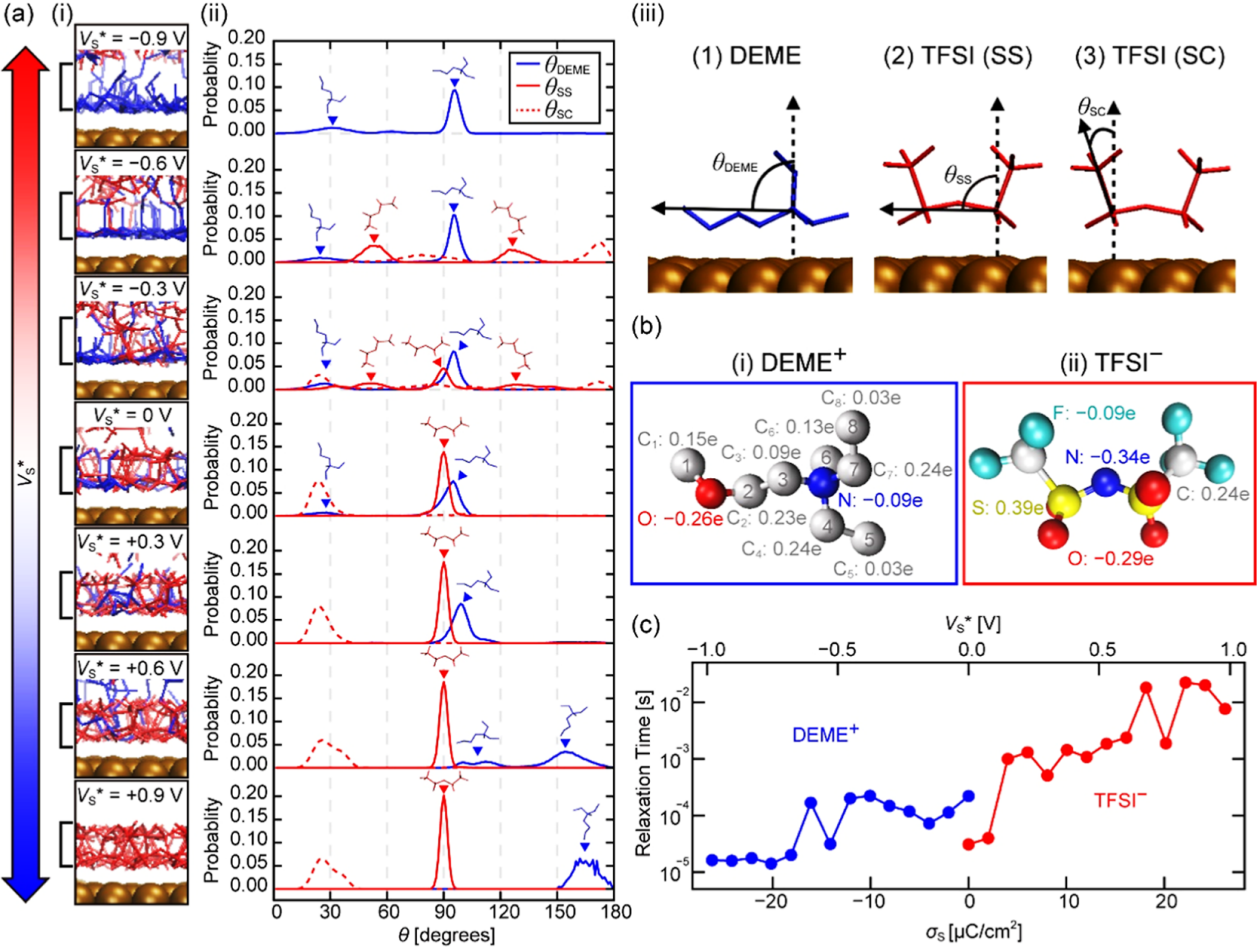
Molecular orientations and stability in the first adsorption layer at the DEME-TFSI/Au(111) interface analyzed with the MD simulation results. (a) (i) Snapshots of the first adsorption layer models and (ii) molecular orientations in them simulated with various *V*_s_*. (iii) Definition of the molecular orientation angles. (b) Charge distribution in (i) DEME^+^ and (ii) TFSI^–^. (c) Relaxation time of the DEME^+^ and TFSI^–^ in the first adsorption layer plotted in the *V*_s_* range of −1 ∼ 0 V and 0 ∼ + 1 V, respectively (plots for the full *V*_s_* range are shown in Figure S7).

For the DEME^+^ ions, they mostly take a flat-lying orientation (θ_DEME_ = ∼100°) at *V*_s_* = −0.9 V. This is because their carbon atoms are all positively charged [Figure 5b­(i)] and hence attracted by the negatively charged substrate. Meanwhile, ∼ 10% of the DEME^+^ ions are oriented with θ_DEME_ = ∼30° to keep the negatively charged oxygen away from the substrate. This orientation distribution remains almost the same within *V*_s_* = −0.9 ∼ 0 V. With increasing *V*_s_* from 0 V to +0.9 V, the flat-lying orientation (θ_DEME_ = ∼100°) gradually changes to the upright orientation (θ_DEME_ = ∼160°) with their negatively charged oxygen oriented to the positively charged substrate.

For TFSI^–^ ions, their oxygen and sulfur atoms respectively have strongly negative or positive charges, producing a large intramolecular dipole as shown in [Figure 5b­(ii)]. Owing to the electrostatic interaction between this dipole and the charged substrate, TFSI^–^ takes a relatively uniform and stable adsorption structure with their oxygen atoms oriented to the substrate at positive *V*_s_*.

This difference in the adsorption stability can be more quantitatively understood by analyzing the ion relaxation time in the first adsorption layer. Figure 5c shows the *V*_s_* dependence of the ion relaxation time. For the definition of the relaxation time, see Figure S7 and its associated texts. At *V*_s_* <0, DEME^+^ adsorbs on the substrate and its relaxation time decreases with decreasing *V*_s_* and saturates to ∼ 10 μs. Meanwhile, at *V*_s_* >0, the first adsorption layer is dominated by TFSI^–^ and its relaxation time increases with increasing *V*_s_* and saturates to ∼ 10 ms. Thus, TFSI^–^ at *V*_s_* = +1 V adsorbs on the substrate is ∼ 1000 times more stable than DEME^+^ at *V*_s_* = −1 V.

This difference in the adsorption stability explains the *V*_s_ dependence of the iso-Δ*f* surface images shown in Figure 4. At *V*_s_ < 0 V, the tip is scanned over the thermally fluctuating adsorbed DEME^+^ ions, so that the obtained AFM images apparently show a blurred molecular-scale contrast with small corrugations. In contrast, at *V*_s_ > 0 V, the tip can precisely trace the corrugations of the firmly adsorbed TFSI^–^ ions, so that the obtained AFM images show a clearer molecular-scale contrast with apparently higher surface roughness.

Comparison between [Figures 5 and 2c­(i)] provides insights into the physical origin of the drastic change at Δ*V*_s_* = 0 V (i.e., *V*_s_* = −0.38 V). The first adsorption layer is dominated by the cations at *V*_s_* < −0.3 V. Meanwhile, when *V*_s_* exceeds this value, the anions start to change their adsorption structure to replace the cations in the first layer [Figure 5a­(ii)]. This significantly increases the thickness of the first adsorption layer and alters the arrangements of the upper IL layers, as seen in [Figure 2c­(i)]. As the origin of the drastic change is the introduction of anions into the first adsorption layer, this voltage does not correspond to the potential of zero charge (i.e., *V*_s_* = 0 V) but is negatively biased. Additionally, the cation does not abruptly disappear at *V*_s_* = 0 V; rather, it gradually decreases up to 1 V while changing its orientation. Therefore, it is reasonable that the contrast becomes gradually clearer.

The molecular ordering in the first adsorption layer affects the layered distribution of the ions above it. At *V*_s_* >0, TFSI^–^ forms a quasi-solid-state adsorption layer whose surface is terminated with fluorine atoms. In general, fluorine-terminated surfaces exhibit a low affinity to water and oil due to their low surface tension. Therefore, a large gap is formed between the first adsorption layer and the ions above it as shown in [Figures 2c­(iii) and S3a]. This gap reduces the effect of the negative charge of the first adsorption layer on the layered ordering of the ions above it. In contrast, at *V*_s_* <0, the first adsorption layer is mostly terminated with the positively charged carbon atoms of DEME^+^, which does not exhibit such water or oil repellency. Thus, TFSI^–^ is attracted to form a distinct second adsorption layer. Similarly, alternating cation and anion layers are formed on it. Therefore, a more distinct layered structure is observed at *V*_s_* <0.

The time spent for scanning across a single molecule in the *x* directions was ∼ 80 ms. These time scales are significantly longer than the relaxation time of DEME^+^, but on the same order for TFSI^–^. Therefore, the TFSI^–^ adsorption layer is more clearly observed than that of DEME^+^ (Figure 4).

### Relationship between Interfacial IL Structure and EDL Capacitance

The differential capacitance (*C*_d_) of EDLCs at the DEME-TFSI/Au(111) interface was measured by electrochemical impedance spectroscopy (EIS). We carried out the EIS at different frequencies (*f*_EIS_) in a range of 1–10,000 Hz with a voltage amplitude of 10 mV. Bode and Nyquist plots at various electrode potentials (*V*_EIS_) are shown in [Figure 6a­(i,ii)], respectively. Here, *C*_d_ is given by the following equation.^87–90^

3 $$C_{d}=\frac{1}{2\pi f_{EIS}Z^{\prime \prime }}$$

where *Z*″ is the imaginary part of the impedance. The obtained *f*_EIS_ dependence of *C*_d_ density is shown in [Figure 6a­(iii)]. This graph shows that the measured *C*_d_ density decreases with increasing *f*_EIS_. The *C*_d_ density versus potential curves measured with *f*_EIS_ = 1, 10, 100, and 1000 Hz are shown in Figure S8. For all the *f*_EIS_ values, “bell-shaped”^91^ *C*_d_ density curves were observed. The following discussions will be made for the *C*_d_ density curve obtained at *f*_EIS_ = 1 Hz [Figure 6a­(iv)] as this condition is closest to the time scale of our AFM measurements.

**Figure 6:**
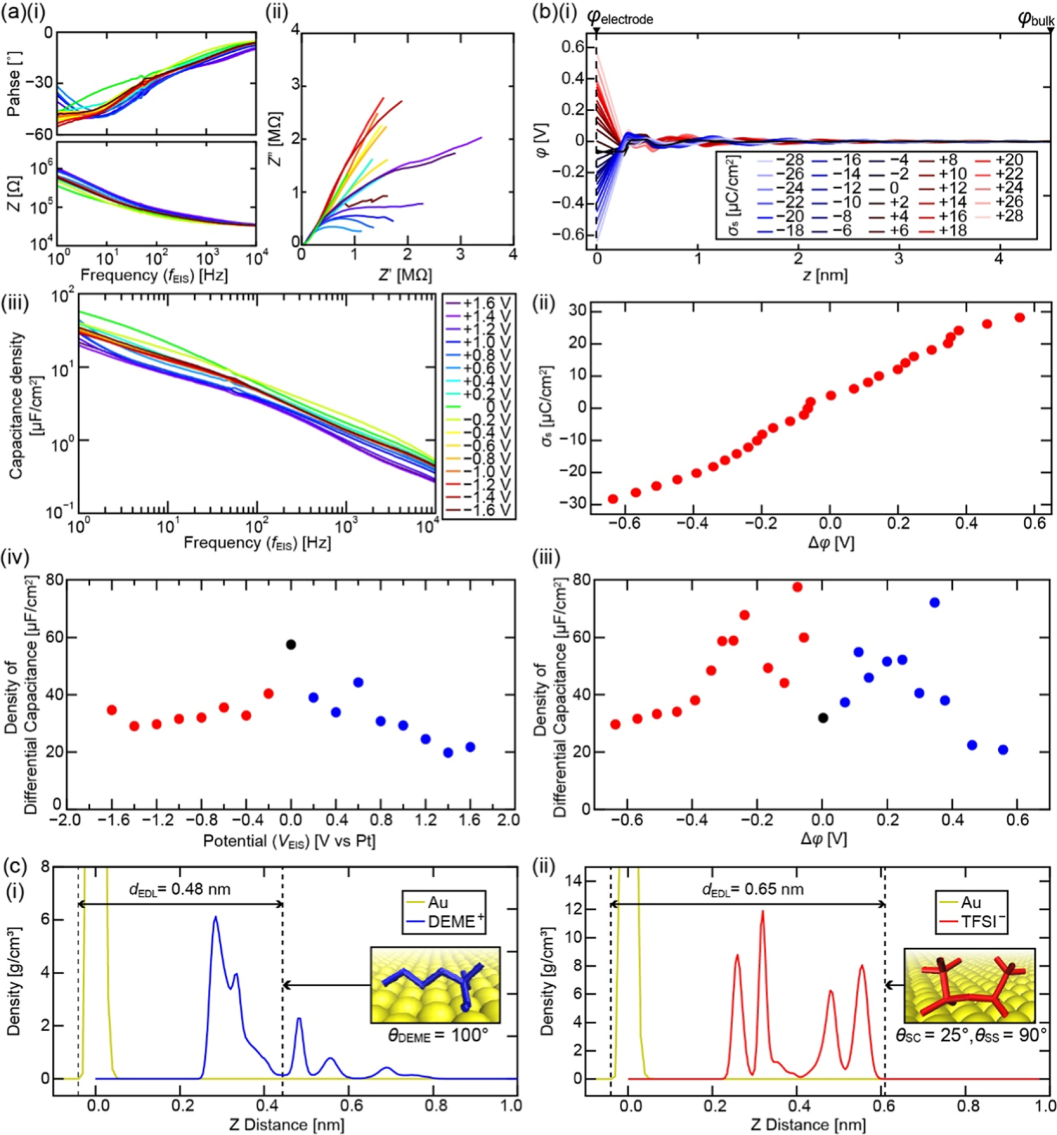
Relationship between capacitance and interfacial IL structure. (a) Experimental results obtained by EIS with different *V*_EIS_. (i) Bode plots. (ii) Nyquist plots. (iii) *f*_EIS_ dependence of the capacitance density estimated from (i). *Z*, *Z*′, and *Z*″ are impedance and its real and imaginary parts, respectively. (iv) *C*_d_ density plotted as a function of *V*_EIS_ at *f*_EIS_ = 1 Hz. (b) MD simulation results. (i) Potential versus *z* curve at different σ_s_. (ii) Δφ dependence of σ_s_. (iii) Δφ dependence of *C*_d_ density. (c) Density profiles along *z* direction of (i) cations at Δφ = −0.56 V and (ii) anions at Δφ = +0.56 V. The major molecular adsorption structures are shown in the insets.

We found that the *C*_d_ density at a high potential (*V*_EIS_ = +1 ∼ + 1.6 V) was higher than that at a low potential (*V*_EIS_ = −1 ∼ −1.6 V). For example, *C*_d_ density at *V*_EIS_ = −1.4 V (∼29.1 μF/cm^2^) is 1.46 times higher than that at *V*_EIS_ = +1.4 V (∼19.9 μF/cm^2^). This ratio is approximately the same as those obtained with *f*_EIS_ = 10, 100, and 1000 Hz (See Figure S8 and Table S2).

The *C*_d_ was also calculated from the MD simulation results. The potential (φ­(z)) distribution along the *z* axis for different σ_s_ was calculated from the simulated charge density distribution with Poisson’s equation as shown in [Figure 6b­(i)]. The voltage drop at the interface (Δφ) is given by the following equation.

4 $$\Delta \varphi =\varphi_{electrode}-\varphi_{bulk}$$

where φ_electrode_ and φ_bulk_ denote φ­(*z*) values at the electrode (*z* = 0 nm) and in the bulk (*z* = 4.5 nm), respectively. [Figure 6b­(ii)] shows Δφ dependence of σ_s_. By differentiating this curve, we obtained the *C*_d_ density curve as shown in [Figure 6b­(iii)].

5 $$\frac{C_{d}}{S}=\frac{\partial \sigma_{s}}{\partial \Delta \varphi }$$

where *S* denotes the surface area of the electrode. The obtained *C*_d_ density curve shows a “camel shape”,^91^ which does not agree with the EIS result shown in [Figure 6a­(iv)].

A previous MD simulation study investigated the dependence of the *C*_d_ density curve on the Au force field.^92^ They reported that the *C*_d_ density curve calculated for the PYR-TFSI/Au(111) interfaces showed a camel shape when a force field with relatively strong interaction of Au atoms (e.g., Heinz model) was used, while bell-shaped *C*_d_ density curve was obtained when a force field with relatively weak interaction (e.g., GolP) was used. In this study, the Heinz model was used for the Au force field, which may account for the disagreement between our experiments and simulation. Thorough studies would be required to fully investigate the effect of Au force field, including the charge and the Lennard-Jones parameter scaling that was not pursued here and more sophisticated metal models such as treating imaging charges. However, it is notable that the asymmetric behavior of *C*_d_ with respect to voltage, a characteristic in this experiment, was well reproduced even when using the Heinz model.

Meanwhile, *C*_d_ at Δφ below −1 V or above +1.5 V reported in the previous study takes almost the same value regardless of the force filed. Therefore, here we focus on *C*_d_ density at a relatively high |Δφ| value. *C*_d_ density at Δφ = −0.56 V (*V*_s_* = −1.08 V) is ∼ 31.61 μF/cm^2^, which is 1.52 times higher than the value ∼ 20.85 μF/cm^2^ at Δφ = +0.56 V (*V*_s_* = +1.08 V). This ratio agrees well with the one obtained with our EIS experiment (∼1.46).

In general, the capacitance of the EDLC (*C*_EDLC_) is given by the following equation.

6 $$C_{EDLC}=\varepsilon \frac{S}{d_{EDL}}$$

where ε is the dielectric constant and *d*_EDL_ is the thickness of the EDL. When ε and *S* are constant, *C*_EDLC_ is inversely proportional to *d*_EDL_. The *d*_EDL_ is often defined by the thickness of the first adsorption layer as the first adsorption layer mostly compensates the electrode surface charge. Thus, we estimated *d*_EDL_ from the MD simulation result.

The orientation distributions of cations at Δφ = −0.56 V and anions at Δφ = +0.56 V in the first layer are shown in Figure S9. These distributions are similar to those at *V*_s_* = ± 0.9 V shown in Figure 5a. At Δφ = −0.56 V, the cations mostly take a flat-lying orientation (θ_DEME_ = ∼ 100°). This orientation should provide a relatively low *d*_EDL_ and hence a high *C*_EDLC_. In the simulation, only the topmost Au layer was charged. Thus, we defined *d*_EDL_ as the distance from the topmost Au layer to the flat-lying cations as shown in [Figure 6c­(i)], and obtained *d*_EDL_ = 0.48 nm. Meanwhile, at Δφ = +0.56 V, all anions take an orientation close to θ_SS_ = ∼ 90° and θ_SC_ = ∼ 25°. By defining *d*_EDL_ in a similar way, we obtained *d*_EDL_ = 0.65 nm as shown in [Figure 6c­(ii)]. Accordingly, *d*_EDL_ at Δφ = +0.56 V is 1.27 times thicker than that at Δφ = −0.56 V. This is consistent with the experimental results, where the thickness of the first adsorption layer with a positive bias [Figure 2b­(iii) and 3f] appears thicker than that with a negative bias [Figures 2b­(ii) and 3e]. These results consistently suggest that the *C*_d_ of an EDLC device can be improved by reducing the thickness of the first adsorption layer. Our results also suggest the critical role of the intramolecular charge distribution in controlling such a molecular adsorption structure and stability.

## Conclusion

In this study, we have measured the DEME-TFSI/Au(111) electrode interface structures with subnanoscale resolution by 3D-SFM with variable tip and sample bias voltages. The 3D images obtained with a positive and negative *V*_t_ revealed the differences in the *V*_s_ dependence of the molecular-scale IL structure. Comparing the experiments and MD simulations, we found that the layered contrasts mainly reflect the distribution of TFSI^–^ because of the larger MW than DEME^+^. In addition, we also found that a positive *V*_t_ provides a clearer layered contrast (Figure 2b) than a negative *V*_t_ (Figure 2a). This is because a rigid TFSI^–^ layer is formed on the Au-coated tip surface with a positive *V*_t_, and hence its electrostatic interaction with the surrounding ions emphasizes the layered contrast. This result clarifies the influence of the tip bias voltage on the measurement of the IL interface structure and the need to control it, improving the methodology for the IL structure analysis by 3D-AFM.

The molecular-scale contrast of the iso-Δ*f* surface image obtained at the first adsorption layer position is clearer at *V*_s_ > 0 V than at *V*_s_ < 0 V (Figure 4). At *V*_s_ > 0 V, the high intramolecular dipole of the anion forms a relatively uniform and highly stable adsorption layer. Thus, the tip can precisely trace the corrugations of the firmly adsorbed TFSI^–^ ions, so that the obtained AFM images show a clearer molecular-scale contrast. Meanwhile, at *V*_s_ < 0 V, DEME^+^ is not strongly polarized and thus forms a thermally fluctuating adsorption structure with a much shorter relaxation time than anions. Consequently, the obtained AFM images show a blurred molecular-scale contrast with small corrugations. These results demonstrate the unique capability of 3D-SFM to visualize ion distribution with subnanoscale resolution simultaneously in both vertical and lateral directions. Such information provides insights into the stability and thickness of the first adsorption layer and the associated device functions such as EDL capacitance.

We further investigated *C*_d_ of the EDLC as a function of electrode potential using EIS and MD simulation and found that *C*_d_ at a negative potential was ∼ 1.5 times higher than that at a positive potential. This is due to the thinner *d*_EDL_ of the adsorbed cations with a flat-lying orientation on the negatively charged electrode. This suggests that *C*_d_ can be improved by reducing the thickness of the first adsorption layer. These findings provide important guidelines for the design of IL devices that exhibit higher capacitance. While asymmetric bias-dependence of *C*_d_ has been previously reported in different IL/solid systems,^87–90^ it has not been observed for the DEME-TFSI/Au(111) interface. In addition, unlike previous studies that rely on interpreting macroscopic EIS measurements through molecular-scale MD simulations, our approach provides molecular-scale real-space experimental evidence that can verify the models predicted by the MD simulation. These methodological advancements not only deepen our understanding of the DEME-TFSI/Au(111) interface but also offer a broadly applicable framework for investigating diverse EDL interface phenomena and their implications for device performance.

## Methods

### Sample Preparations

DEME-TFSI (>99.0%) and Au (99.99+%) were purchased from Kanto Chemical Co. and Niraco Co., respectively. The Au(111) substrate was prepared by depositing a 200 nm Au film on cleaved mica with a vacuum evaporation system (KE604TT-KFH4, K’s Tech). For the 3D-SFM and cyclic voltammetry measurements, the center of the mica was masked with a 1 mm wide tantalum plate during the deposition to fabricate two isolated Au electrodes. The two electrodes were wired with copper wire and sealed with silicone rubber to prevent the wiring area from contacting the IL. The area of one Au electrode in contact with the IL was 0.3075 cm^2^. For the EIS measurements, an Au-coated mica substrate with 1 mm width and 10 mm length was fabricated using the method described above. Then, the substrate was wired with a copper wire, and the wiring area was sealed with silicone rubber. The area of the Au exposed to the IL was 1 mm^2^. Pt (99.95%, Niraco Co.) was used as counter and reference electrodes. The area of the counter electrode immersed in the IL was 470 mm^2^, which is sufficiently larger than that of the Au electrode. Before cyclic voltammetry, 3D-SFM, and EIS measurements, water contained in the sample was removed by drying under a vacuum environment for 24 h.

#### 3D-SFM Measurements

The 3D-SFM measurements were performed using a custom-built 3D-SFM with an ultralow-noise cantilever deflection sensor^93,94^ and a highly stable photothermal cantilever excitation system.^95,96^ A commercially available phase-locked loop circuit (OC4, SPECS) was used for oscillating a cantilever at its *f*_0_ with constant amplitude (*A*) and for detecting Δ*f* induced by the force variation. The 3D-SFM was controlled by a commercially available controller (ARC2, Oxford Instruments) with a modification in the software. The tip was vertically scanned with a fast sinusoidal wave, whereas it was slowly scanned in the lateral direction. During the tip scan, Δ*f* induced by the force variation was recorded to produce a 3D Δ*f* image. Meanwhile, the tip–sample distance was continuously regulated such that the average Δ*f* was equal to a set point value. Thus, a 2D height image and a 3D Δ*f* image were simultaneously obtained. All 3D-SFM images were obtained with a pixel size of 128 × 128 × 256 pix^3^, a *z* modulation frequency of 195.3 Hz, and an imaging time of 218 s. The approach curves were used to construct the 3D Δ*f* images. The 3D-SFM images were obtained using an AC55 cantilever from Olympus. The spring constant (*k*), quality factor (*Q*), and *f*_0_ of this cantilever in DEME-TFSI are 33.14 N/m, 1.62, and 834 kHz, respectively. Figure S10 in the Supporting Information shows the frequency spectra of cantilever Brownian motion measured in DEME-TFSI and amplitude vs frequency curves measured with photothermal excitation in DEME-TFSI.

The tip was coated with 5 nm Cr and 30 nm Au films using a sputter coater (KST-CSPS-KF1, K’s Tech) to obtain a conductive AFM tip. *V*_s_ and *V*_t_ were produced with a function generator (WF1974, NF Co.) 3D-SFM images were obtained with (1) a constant *V*_s_ at −1 V, (2) *V*_s_ sweep from −1 V to +1 V, (3) a constant *V*_s_ at +1 V, (4) *V*_s_ sweep from +1 V to −1 V, and (5) constant *V*_s_ at –1 V. These five 3D images were recorded at *V*_t_ = –0.5 V and +0.5 V. Thus, 10 images were recorded in total. The *yz* cross sections of all these 3D images averaged in the *x* direction are presented in Figure S5.

#### Electrochemical Measurements

Cyclic voltammetry measurements were performed with samples prepared in the same way as we did for the 3D-SFM experiments. The voltage applied to the sample was produced with a function generator (WF1974, NF Co.), and the current was measured with a homemade *I*–*V* amplifier using an operational amplifier (OPA627BP, Texas Instruments). The data was obtained with a data logger (ZR-RX40, OMRON). The voltage was swept from −4 V to +4 V at 10 mV/s. The EIS measurements were carried out with a frequency response analyzer (FRA)­(FRA5097f, NF Co.) and a potentiostat (HZ-5000, HOKUTO DENKO). The frequency of the FRA output was swept from 1 Hz to 10 kHz and its amplitude was fixed at 10 mV. The electrochemical potential of the sample with respect to the Pt reference electrode was varied from −1.6 V to +1.6 V with a 0.2 V step using the potentiostat. After setting the potential, we waited for approximately 20 min before performing the EIS measurement. The impedance was calculated by dividing the applied voltage by the current obtained with the FRA. The phase delay was also measured with the FRA.

#### MD Simulations

The simulated system was composed of 2000 DEME^+^, 2000 TFSI^–,^ and 2 plates of the Au substrate as negative and positive electrodes (Figure 1g). The total number of particles in the system was 56,912. The united-atom force field of DEME^+^ was developed as described later. The Borodin & Smith^97^ model was employed for TFSI^–^. The Heinz model^98^ was used for Au since it was known that the Heinz model is suitable for the simulation of ionic liquids at room temperature on the Au surface.^92^

The MD simulation was performed under the constant volume and temperature (300 K) condition, using the Berendsen thermostat.^99^ The employment of the united-atom model enabled us to use a time step of 2 fs. The periodic boundary condition was imposed, and long-range interactions were calculated by the particle mesh Ewald method^100^ with a 12 Å cutoff in real space. All MD simulations were performed by using AMBER18.^101^ The Au plate consists of three layers with an fcc Au structure and the *x* and *y* dimensions of 8.413 and 8.196 nm, respectively, which were the *x* and *y* dimensions of the simulation box. The bulk DEME-TFSI solution was sandwiched by two Au plates with their (111) surfaces facing to the ionic liquid. The positions of Au atoms were constrained by a harmonic potential with a force constant of 60 kcal/mol/Å^2^. The Au plates were initially set at some distance, and the distance was gradually shortened until the bulk density of the DEME-TFSI solution reached the experimental value (1.41 g/cm^3^). The resultant distance between plates (center-to-center distance between two Au layers that directly face to the ionic liquids) was 14.753 nm. The box size in the *z* direction was set at the maximum value (50 nm) to minimize the effect of periodic boundary conditions in this direction. To impose the electric fields, one layer of Au substrate directly facing to the ionic liquid was assumed to be uniformly charged.^102^ Placing a charge of 121.3e on an area of 8.413 × 8.196 nm^2^ resulted in 26.21 μC/cm^2^. Accordingly, 0.0978e (=121.3*e*/1152 atoms per one layer) was placed on the Au atoms in the positive electrode and −0.0978e in the negative electrode. The MD simulation was performed for 1 μs under constant volume and temperature (300 K) conditions. The trajectories were saved every 10 ps and the last 500 ns trajectory (i.e., 50,000 configurations of DEME^+^ and TFSI^–^) was used in the analysis.

To develop the force field of DEME^+^, we followed the paper by Siqueira and Ribeiro.^103^ It developed the force field of *N*-ethyl-*N*,*N*-dimethyl-*N*-(2-methoxyethyl)­ammonium (MOENM_2_E), a molecule in which an ethyl group in DEME is replaced by a methyl group, based on OPLS force field. OPLS force fields were used for bonds, angles, and dihedral angles, and all the parameters are written in the supplementary file (parmIL.dat, which also includes the Lennard-Jones parameters and those of TFST^–^ and Au). The charges on particles were derived from electrostatic potential obtained by ab initio calculations using Gaussian09 ^104^ at the MP2 level with a 6–311+G* basis set, and hydrogen charges were summed into heavy atoms. However, a well-known problem is that the developed parameters of ionic liquid molecules in this usual way do not reproduce thermodynamic characters such as density and diffusion coefficient.^102,103^ To address this issue, we employed the electronic continuum correction concept,^105^ which scales down the charges on ionic liquids to account for the polarization in nonpolarizable models. The σ_s_ parameters in the Lennard-Jones potential also must be scaled along the charge scaling.^106^ Several sets of scaling parameters for charges and Lennard-Jones potential were examined to find a suitable set that simultaneously reproduces density and diffusion coefficient (Figure S11). It was found that the experiments at 300 K and 1 bar were well reproduced when charges were scaled by 0.80 and parameters of sigma in the Lennard-Jones potential were scaled by 1.07. Note that σ_s_ parameters in the parmIL.dat file have already been scaled. The simulated temperature dependency of the diffusion coefficient and density reproduced the experiment^107,108^ well (Figure S12) even using the scaling factor determined at 300 K and 1 bar. The dielectric constant computed from 100 ns simulation was 3.5. The scaled charges and atom type of DEME^+^ and TFSI^–^ are shown in Figure S13, and the library files in AMBER format were provided as supplementary electronic files (DEME.lib and TFSI.lib).

## Supplementary Material





## Notes

Library files in AMBER format (DEME.lib and TFSI.lib) (ZIP)
Relationship between *V*_s_* and σ_s_ in the MD simulation (Figure S1). Relationship between *V*_s_ and σ_s_ in the experiment (Figure S2) σ_s_ dependence of snapshot of the simulation model and the total ion density distribution in the MD simulation (Figure S3). Δ*f* and force versus distance curves before and after force conversions (Figure S4). *yz* cross sections of the 3D-SFM images averaged in *x* direction at difference *V*_t_ and *V*_s_ (Figure S5). Magnified view of *xz* cross sections derived from the 3D-SFM images (Figure S6). Relaxation time of DEME^+^ and TFSI^–^ in the first adsorption layer in the MD simulation (Figure S7). Frequency dependence of *C*_d_ measured by EIS (Figure S8). Orientations of the ions adsorbed on the Au electrode at Δφ = ± 0.56 V (Figure S9). Deflection noise density spectrum of the cantilever and amplitude versus frequency curve measured in DEME-TFSI (Figure S10). Dependence of densities and diffusion coefficients on the scaling factors for charges and the σ_s_ parameters in the Lennard-Jones potential (Figure S11). Dependence of the diffusion coefficient and density on temperature (Figure S12). Scaled charges and atom type of DEME^+^ and TFSI^–^ (Figure S13) (PDF)
