# Supplementary material for: Visualizing Molecular-Scale 3D Distributions of Ionic Liquids in Electric Double-Layer Capacitor by 3D Scanning Force Microscopy with Variable Tip/Sample Bias Voltages
Source: ACS Appl Mater Interfaces. 2025 Sep 6;17(37):52868–82. doi: 10.1021/acsami.5c11718 (PMC12447403; doi:10.1021/acsami.5c11718)
Supplement: Supplementary file 2 [file am5c11718_si_002.pdf]

Supporting Information

Visualizing Molecular-Scale 3D Distributions of Ionic Liquids in Electric Double-Layer Capacitor by 3D Scanning Force Microscopy with Variable Tip/Sample Bias Voltages

Takahiko Ikarashi[1,#], Takashi Sumikama[2,#], Kaito Hirata[3], Ryo Sakakibara[4], Takumi Yoshino[4], Kazuki Miyata[1, 2, 4, 5], Keisuke Miyazawa[2, 4, 5], Sunao Shimizu[6], Yoshihiro Iwasa[7, 8*] and Takeshi Fukuma[1, 2, 4, 5*]

[1]Division of Nano Life Science, Kanazawa University, Kakuma-machi, 920-1192 Kanazawa, Japan

[2]Nano Life Science Institute (WPI-NanoLSI), Kanazawa University, Kakuma-machi, 920-1192 Kanazawa, Japan,

[3]Department of Electronics Engineering, Nagoya University, Furo-cho, Chikusa-ku, 464-8603 Nagoya, Japan

[4]Division of Electrical Engineering and Computer Science, Kanazawa University, Kakuma-machi, 920-1192 Kanazawa, Japan,

[5]Division of Frontier Engineering, Kanazawa University, Kakuma-machi, 920-1192 Kanazawa, Japan,

[6]Central Research Institute of Electric Power Industry, 2-6-1, Chozaka, Yokosuka, 240-0196 Kanagawa, Japan,

[7]Deptment of Engineering, The University of Tokyo, 7-3-1, Hongo, Bunkyo-ku, 113-8654 Tokyo, Japan,

[8] RIKEN Center for Emergent Matter Science (CEMS), 2-1, Hirosawa, Wako, 351-0198 Saitama, Japan

[*]To whom corresponding may be addressed.

[#]These authors contributed equally to this work.

Corresponding Author Information:

Yoshihiro Iwasa: iwasay@riken.jp

Takeshi Fukuma: fukuma@staff.kanazawa-u.ac.jp

Relationship between V_s* and σ_s in the MD simulation

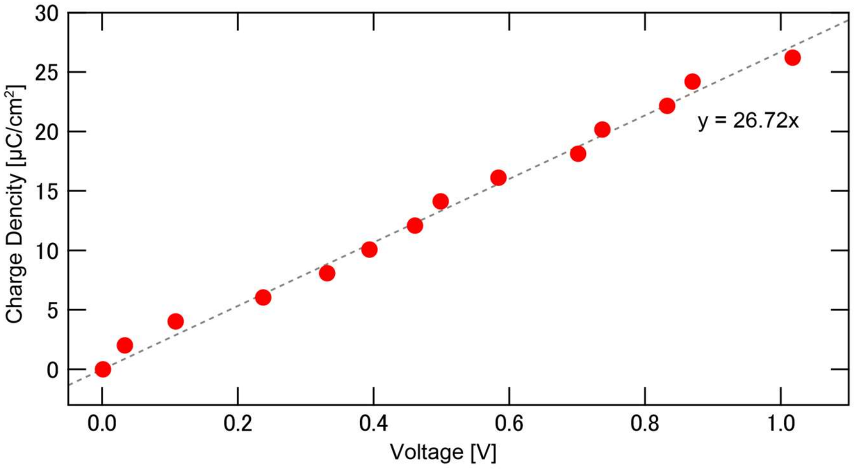

Figure S1: Relationship between the voltage applied between the two opposing electrodes (V_s*) and the absolute value of the surface charge density at the electrodes (σ_s) in the MD simulation.

The differential capacitance (C_d) was estimated by fitting a linear function to the simulated σ_s versus V_s* curve. As shown in Figure S1, the slope of the curve, corresponding to C_d, was estimated to be 26.72 μF/cm^2. Therefore, when 1 V was applied between the two electrodes, 26.72 μC/cm^2 surface charges should be accumulated at the electrode.

Relationship between V_s and σ_s in the experiment

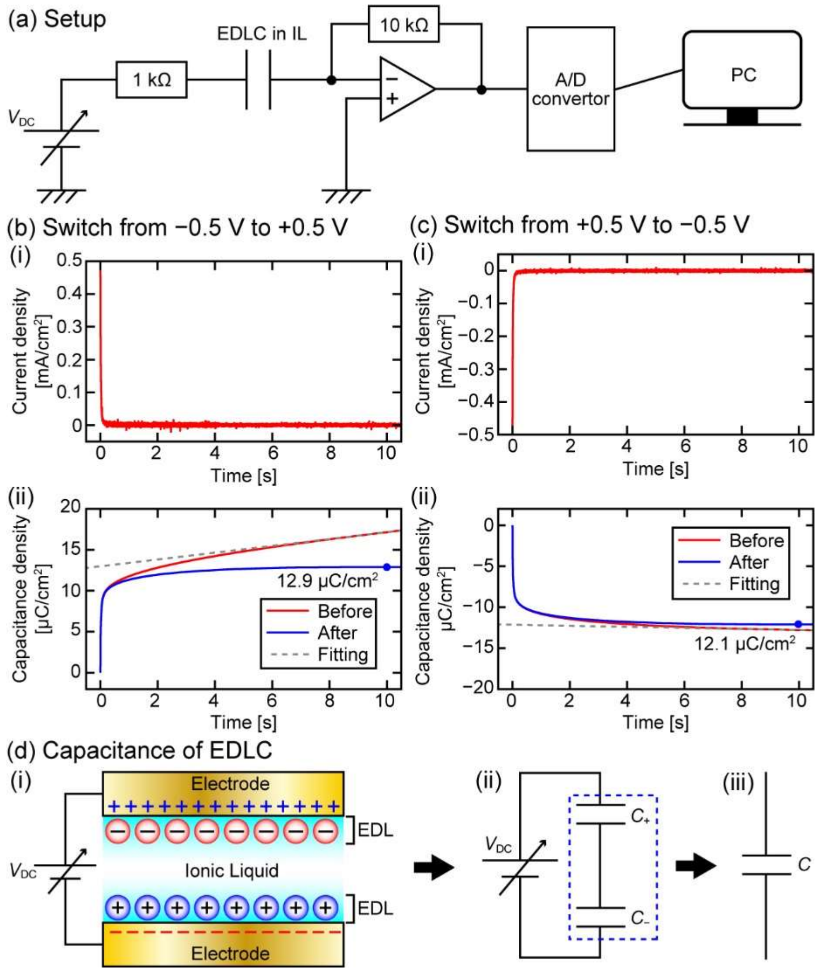

Figure S2: Capacitance measurement of the EDLC formed at the DEME-TFSI/Au(111) interface. (a) Measurement setup. Time lapse changes of (i) the electrode current and (ii) capacitance density recorded after changing the DC bias voltage (V_{DC}) from (b) −0.5 V to +0.5 V and (c) +0.5 V to −0.5 V.

Figure S2a shows the setup for the capacitance measurement. The sample was the same as used in the 3D-SFM measurement. The capacitance part in the circuit diagram in Figure S2a corresponds to the EDLC. A resistor (1 kΩ) is connected in series with this EDLC to make an RC circuit. Transient current was measured after switching V_{DC} from −0.5 V to +0.5 V or from +0.5 V to −0.5 V. To start the measurement after the current is stabilized, the voltage change was performed after at least one hour since the initial voltage (−0.5 V or +0.5 V) was applied. The current was converted from current to voltage by an I-V converter and then converted to digital values by an AD converter (NI USB-6259, National Instruments) and recorded by a PC.

The time dependence of the current density is shown in Figure S2b-c(i). In these graphs, 0 s corresponds to the time when the applied DC voltage was switched from −0.5 V to +0.5 V or from +0.5 V to −0.5 V. The capacitance density was calculated from these current density values using the following equation.

$$\frac{C}{S} = \frac{Q}{VS} = \frac{1}{V}\int \frac{i}{S}dt$$

The time dependence of the calculated capacitance density is shown in Figure S2b-c(ii). In general, the capacitance density of an EDLC with IL is several μC/cm^2 ~ several tens of μC/cm^2, thus the time constant of the RC circuit should be several ms ~ several tens of ms when if a resistance of 1 kΩ is used. However, from Figures S2b-c(ii), the capacitance did not become constant even at 10 s after switching. This indicates the existence of a constant leakage or electrochemical reaction current. To eliminate the contribution from them, a linear fitting was performed in the range of 9 ~ 10 s and subtracted the fitted curve from the raw data, as shown in Figure S2b-c(ii). The capacitance density as a function of time after subtraction is shown by the blue line in Figure S2b-c(ii). The values at 10 s were adopted, where capacitance density is almost constant. The results are shown in the following Table S1. Here, switching from −0.5 V to +0.5 V (Figure S2b) and from +0.5 V to −0.5 V (Figure S2c) were performed two times for each.

Table S1: Capacitance density of the EDLC

DC Voltage [V]	−0.5 V → +0.5	+0.5 V → −0.5	−0.5 V → +0.5	+0.5 V → −0.5
Capacitance Density[μC/cm^2]	12.9	12.1	12.8	11.5

This table shows that the average value is 12.325 μC/cm^2. Here, the measured capacitance is equivalent to the total capacitance of two equivalent capacitors connected in series as shown in Figure S2d. Therefore, the EDL capacitance density at one electrode surface should be twice larger and hence 24.65 μC/cm^2.

σ_s dependence of the snapshots of the simulation model and the total ion density distribution in the MD simulation

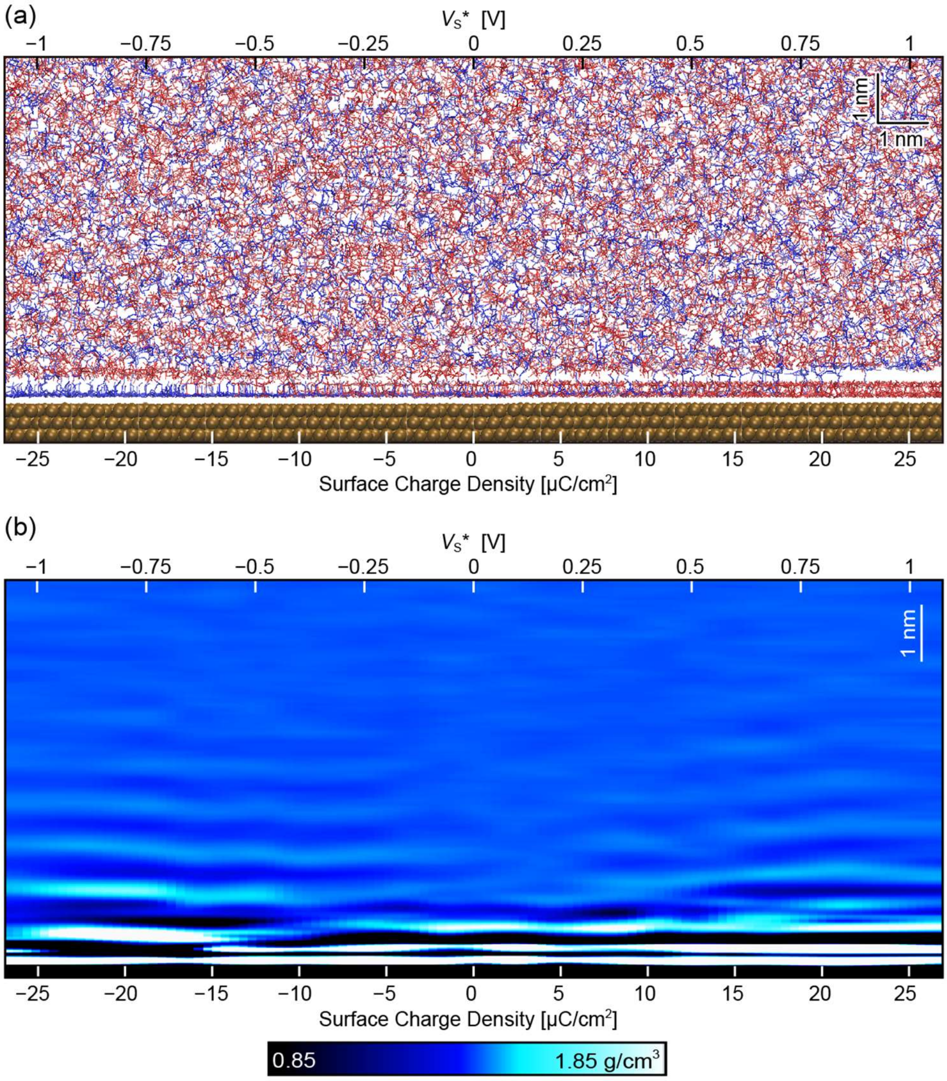

Figure S3: (a) σ_s (V_s^*) dependence of the snapshots of the MD simulation. (b) σ_s (V_s^*) dependence of the total ion density distribution averaged in the direction perpendicular to the figure.

MD simulations were performed with Au surfaces with different charges ranging from −26 μC/cm^2 to +26 μC/cm^2, incremented by 2 μC/cm^2. Parts of snapshot at each surface charge were arranged continuously to create Figure S3. Figure 2c(i) and 2d(i) in the main text show those in the range from −24 μC/cm^2 to +4 μC/cm^2 in Figure S3a and S3b, respectively.

Δf and force versus distance curves before and after force conversions

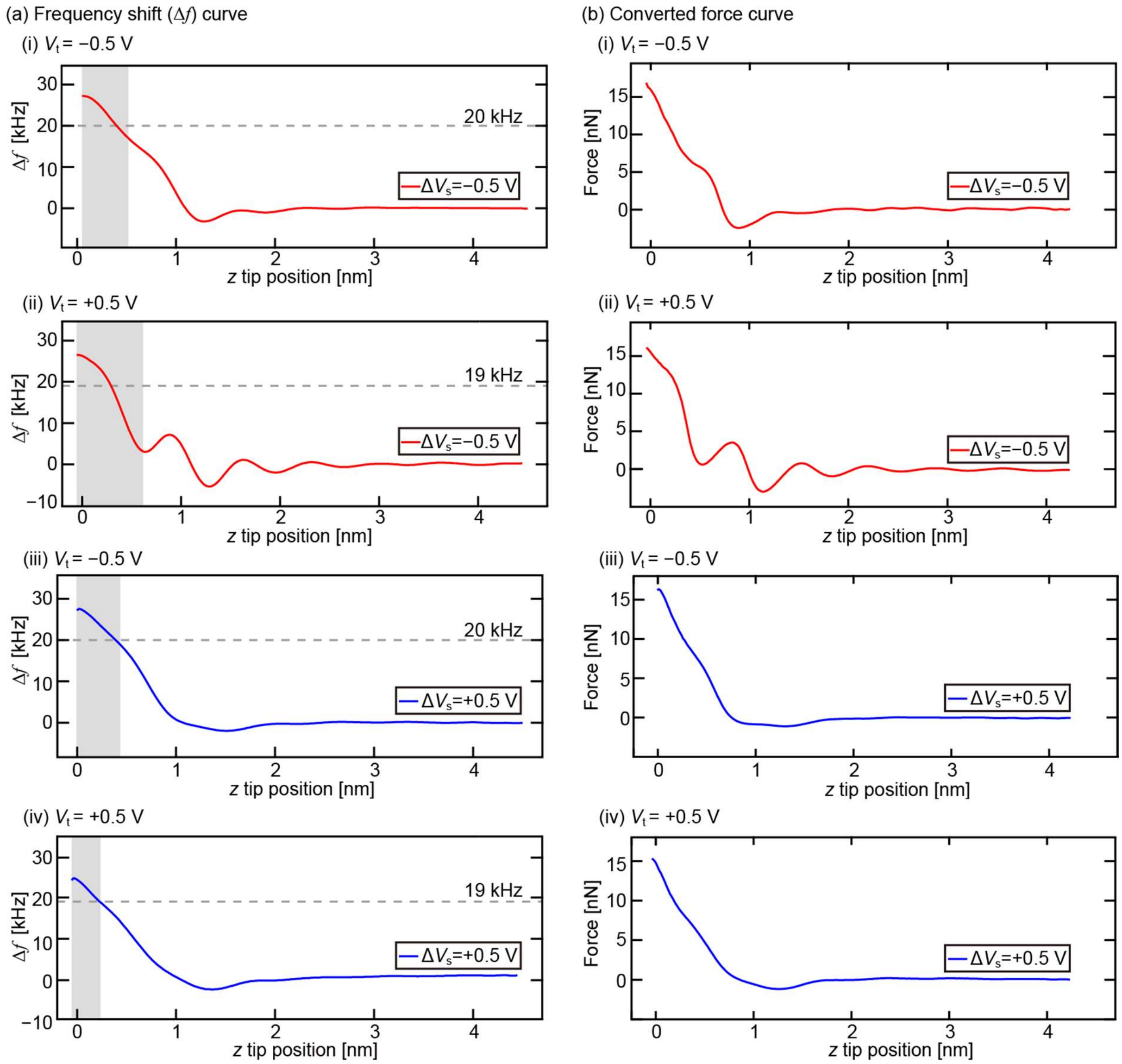

Figure S4: (a) Frequency shift curves. (b) Force curves. (i) $V_t = -0.5$ V, $\Delta V_s = -0.5$ V. (ii) $V_t = +0.5$ V, $\Delta V_s = -0.5$V. (iii) $V_t = -0.5$ V, $\Delta V_s = +0.5$V. (iv) $V_t = +0.5$ V, $\Delta V_s = +0.5$V.

yz cross-sections of the 3D-SFM images averaged in *x* direction at difference V_t and V_s

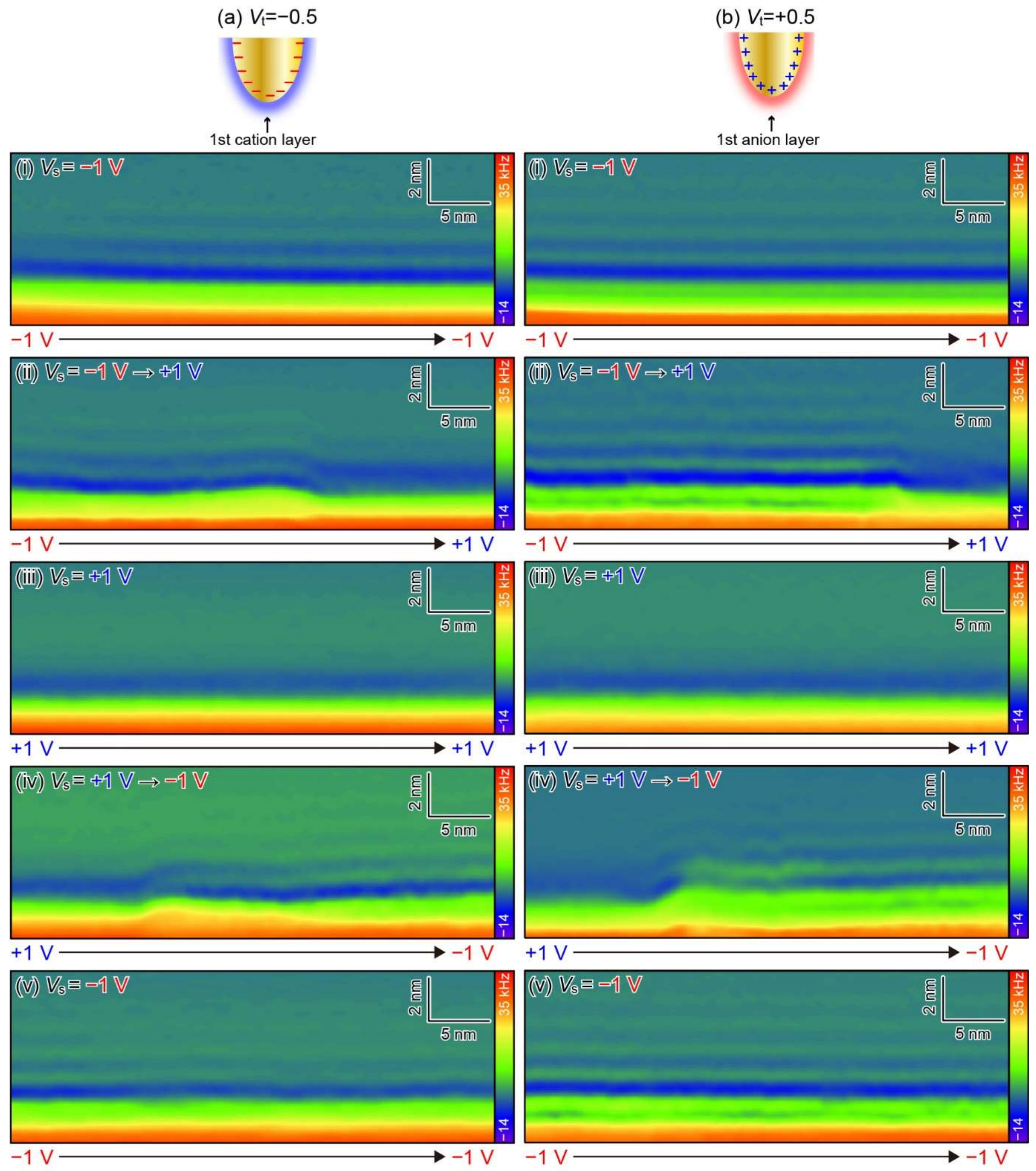

Figure S5: *yz* cross-sections averaged in *x* direction obtained with different V_s and V_t. (a) V_t = −0.5 V (b) V_t = +0.5 V. (i) V_s = −1 V. (ii) V_s was swept from −1 V to +1 V. (iii) V_s = +1 V. (iv) V_s was swept from −1 V to −1 V. (v) V_t was swept from −1 V to +1 V. (vi) V_t was swept from −1 V to +1 V. (iii) V_s = +1 V. (iv) V_s was swept from +1 V to −1 V. (v) V_s = −1 V.

Magnified view of *xz* cross-sections derived from the 3D-SFM images

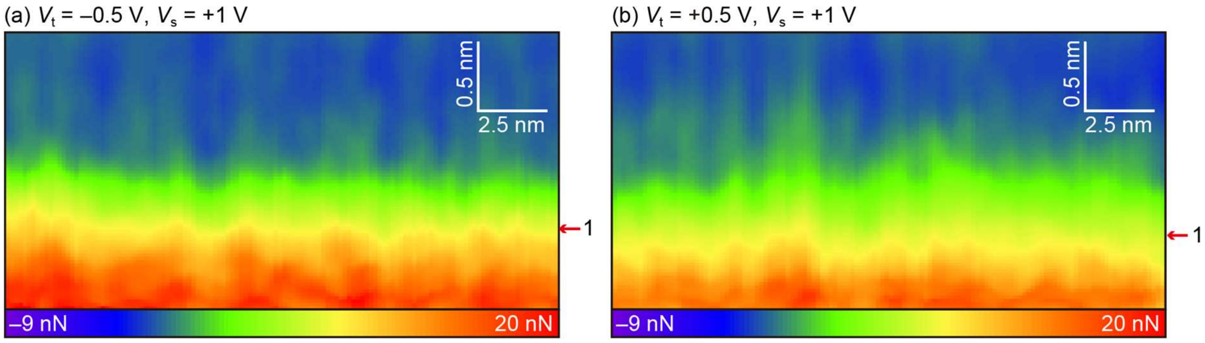

Figure S6: Magnified view of the *xz* cross-sections derived from the 3D-SFM images of DEME-TFSI/Au(111) interface. The 3D-SFM images analyzed here are exactly the same data used for preparing Figures 2 and 4. (a) V_t = −0.5 V, ΔV_s = +1 V. (b) V_t = +0.5 V, ΔV_s = +1 V. The arrows indicate the vertical positions of the force peaks corresponding to the first adsorption layer. These positions are the same as those identified in Figure 3 and those shown in Figure 2.

These magnified *xz* cross-sectional views clearly reveal a non-uniform distribution of force contrast within the first layer, highlighting subtle corrugations that reflect the underlying molecular arrangements in this layer.

Relaxation time of $DEME^+$ and $TFSI^-$ in the first adsorption layer in the MD simulation

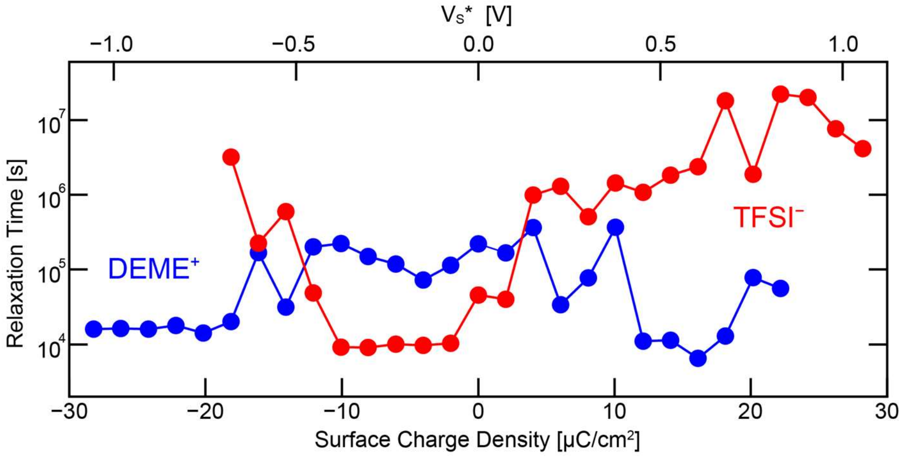

Figure S7: Relaxation time of the $DEME^+$ and $TFSI^-$ conformations in the first adsorption layer in the simulation. The relaxation time for $DEME^+$ and $TFSI^-$ were plotted in the range $V_s^* < 0$ V ($\sigma_s < 0$ μC/cm^2) and $V_s^* < 0$ V ($\sigma_s < 0$ μC/cm^2), respectively.

The orientation of $DEME^+$ is defined by the vector from nitrogen to carbon atom at the end of the longest chain. The orientation of $TFSI^-$ is defined by a vector from one sulfur to another sulfur atom. The correlation functions ($C(t)$) were calculated via the following equation when molecules were persistently attached to the Au surface for 200 ns:

$$C(t) = \frac{\langle \boldsymbol{v}(t)\boldsymbol{v}(0) \rangle}{\langle \boldsymbol{v}(0)\boldsymbol{v}(0) \rangle}$$

where $\boldsymbol{v}$(t) is the vector at time t. The relaxation times were estimated by fitting $C(t)$ from 50 ns to 100 ns via $Ae^{-t/\tau}$, where A is a fraction of the component and τ is the relaxation time.

Frequency dependence of C_d measured by EIS

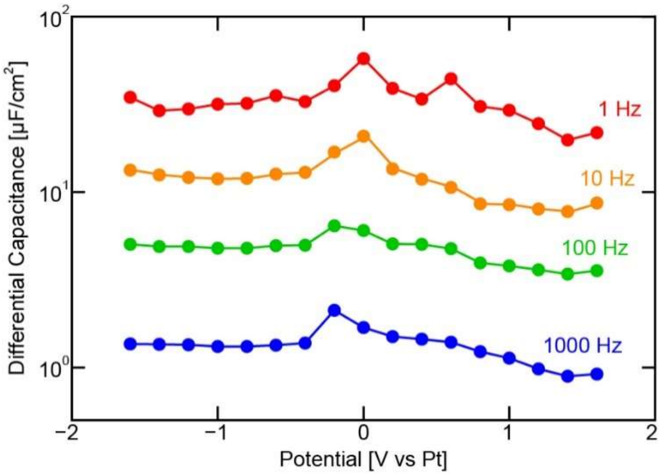

Figure S8: Frequency dependence of differential capacitance density (C_d) obtained by EIS.

Table S2: C_d density at −1.4 V ($C_{-1.4}$) and at +1.4 V ($C_{+1.4}$) at different frequencies and their ratio.

Frequency [Hz]	$C_{-1.4}$ [μC/cm²]	$C_{+1.4}$ [μC/cm²]	Ratio ($C_{-1.4}/C_{+1.4}$)
1	29.1	19.9	1.46
10	12.5	7.8	1.62
100	4.9	3.4	1.43
1000	1.4	0.9	1.52

Orientations of the ions adsorbed on the Au electrode at $\Delta\varphi = \pm 0.56$ V

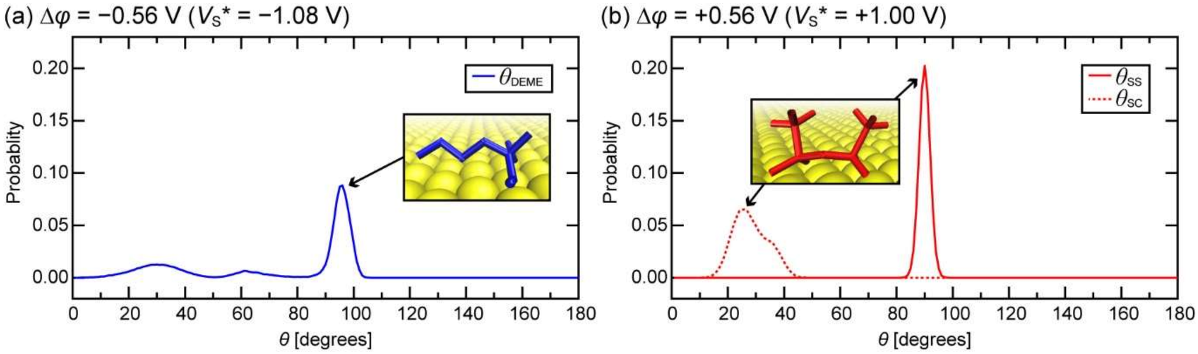

Figure S9: (a, b) Angular distribution of (a) cations at $\Delta\varphi = -0.56$ V and (b) anions at $\Delta\varphi = +0.56$ V. Inset snapshots show the major molecular orientations.

Deflection noise density spectrum of the cantilever and amplitude versus frequency curve measured in DEME-TFSI

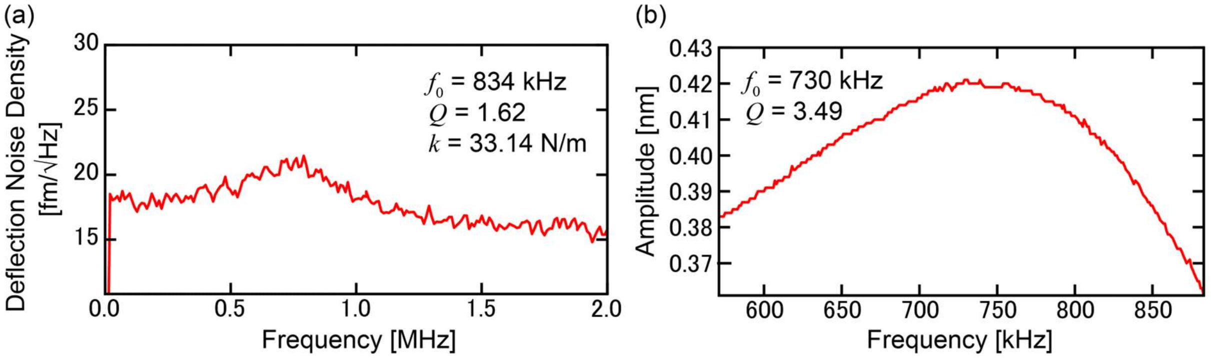

Figure S10: (a) Deflection noise density spectrum of the AC55 cantilever (Olympus) measured in DEME-TFSI. (b) Amplitude versus frequency curve measured with the photothermal excitation method in DEME-TFSI.

Dependence of densities and diffusion coefficients on the scaling factors for charges and the σ parameters in the Lennard-Jones potential

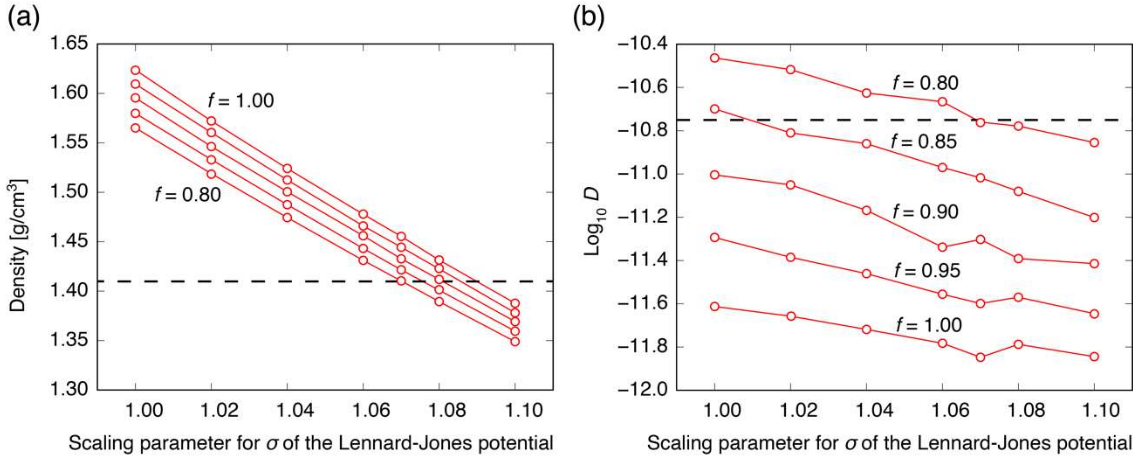

Figure S11: (a) is for densities and (b) is for diffusion coefficient. *D* in (b) is the diffusion coefficient in m^2/s. Dashed black lines shows the experimental values. *f* is the scaling factor for charges. $f = 0.80$ and 1.00 are shown for the clarity in (a). Densities were estimated from the MD simulations of 1000 $DEME^+$ and 1000 $TFSI^-$ system under constant pressure (1 bar) and temperature (300K) conditions with the Berendsen barostat and thermostat. Diffusion coefficients were computed by linear fits for the mean square displacement from 1 ns to 5 ns. From these figures, $f = 0.80$ and the scaling factor for $\sigma = 1.07$ are found to be the best set pair to reproduce experiments.

Dependence of the diffusion coefficient on temperature

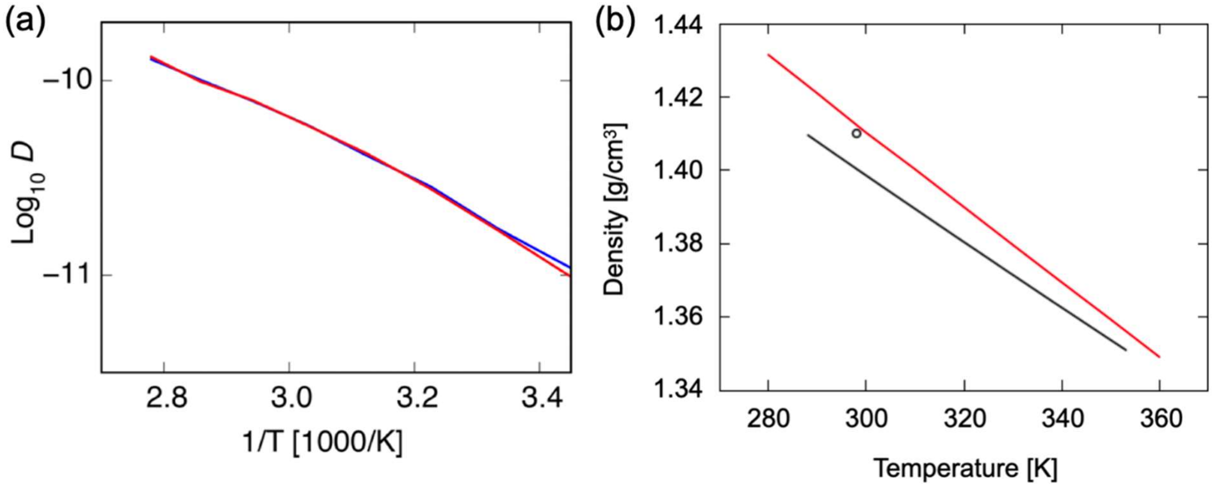

Figure S12: Dependency of the diffusion coefficient and density on temperature. (a) Diffusion coefficient against the inverse of temperature. Blue line indicates the diffusion coefficient of $DEME^{+}$, and red one is for $TFSI^{-}$. (b) Density against the temperature. Black line is the density measured by experiments (ref. 108), and red one was computed here. Black circle indicates the density obtained by another measurement (ref. 109), which was used as a target to be reproduced. The MD simulations were performed at 280, 290, 300, 310, 320, 330, 340, 350, and 360K using a best pair of scaling factors.

Scaled charges and atom type of DEME^{+} and TFSI^{-}

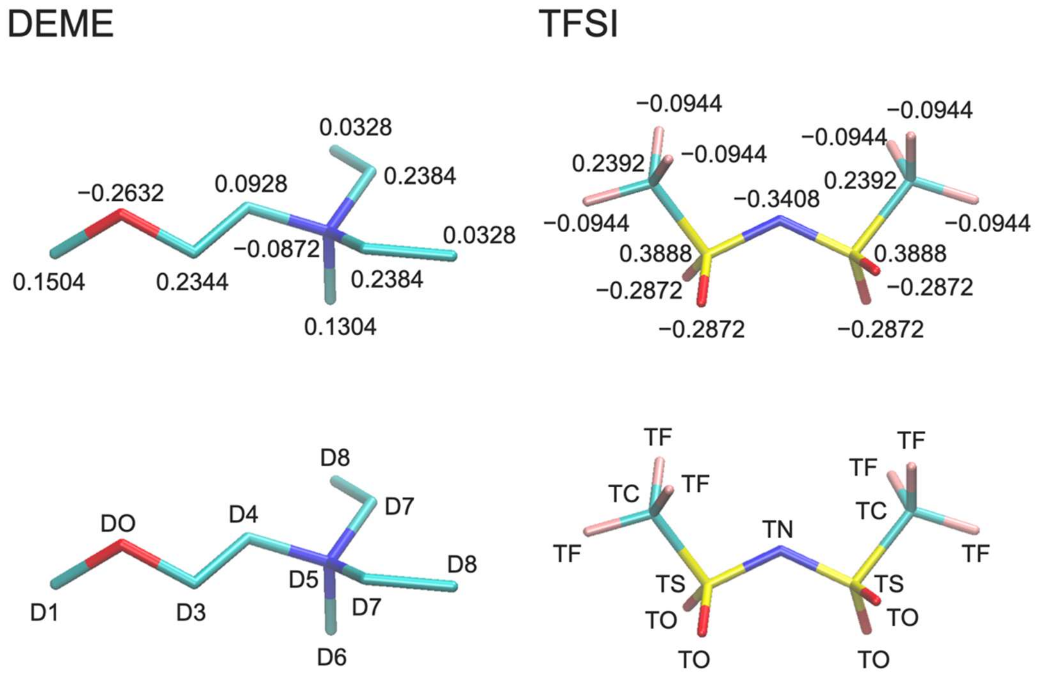

Figure S13: The upper and lower figures show the charges and types, respectively. The carbon, oxygen, nitrogen, sulfur, and fluorine atoms are depicted in cyan, red, blue, yellow, and pink, respectively.
